# Procalcitonin Guidance to Reduce Antibiotic Treatment of Lower Respiratory Tract Infection in Children and Adolescents (ProPAED): A Randomized Controlled Trial

**DOI:** 10.1371/journal.pone.0068419

**Published:** 2013-08-06

**Authors:** Gurli Baer, Philipp Baumann, Michael Buettcher, Ulrich Heininger, Gerald Berthet, Juliane Schäfer, Heiner C. Bucher, Daniel Trachsel, Jacques Schneider, Muriel Gambon, Diana Reppucci, Jessica M. Bonhoeffer, Jody Stähelin-Massik, Philipp Schuetz, Beat Mueller, Gabor Szinnai, Urs B. Schaad, Jan Bonhoeffer

**Affiliations:** 1 Department of Pediatrics, University Basel, Basel, Switzerland; 2 University Children's Hospital Basel, Basel, Switzerland; 3 Department of Pediatrics, Kantonsspital Aarau, Aarau, Switzerland; 4 Basel Institute for Clinical Epidemiology and Biostatistics, University Hospital Basel, Basel, Switzerland; 5 Department of Internal Medicine, University Hospital Basel, Basel, Switzerland; 6 Department of Internal Medicine, Kantonsspital Aarau, Basel, Switzerland; Menzies School of Health Research, Australia

## Abstract

**Background:**

Antibiotics are overused in children and adolescents with lower respiratory tract infection (LRTI). Serum-procalcitonin (PCT) can be used to guide treatment when bacterial infection is suspected. Its role in pediatric LRTI is unclear.

**Methods:**

Between 01/2009 and 02/2010 we randomized previously healthy patients 1 month to 18 years old presenting with LRTI to the emergency departments of two pediatric hospitals in Switzerland to receive antibiotics either according to a PCT guidance algorithm established for adult LRTI or standard care clinical guidelines. In intention-to-treat analyses, antibiotic prescribing rate, duration of antibiotic treatment, and number of days with impairment of daily activities within 14 days of randomization were compared between the two groups.

**Results:**

In total 337 children, mean age 3.8 years (range 0.1–18), were included. Antibiotic prescribing rates were not significantly different in PCT guided patients compared to controls (OR 1.26; 95% CI 0.81, 1.95). Mean duration of antibiotic exposure was reduced from 6.3 to 4.5 days under PCT guidance (−1.8 days; 95% CI −3.1, −0.5; P = 0.039) for all LRTI and from 9.1 to 5.7 days for pneumonia (−3.4 days 95% CI −4.9, −1.7; P<0.001). There was no apparent difference in impairment of daily activities between PCT guided and control patients.

**Conclusion:**

PCT guidance reduced antibiotic exposure by reducing the duration of antibiotic treatment, while not affecting the antibiotic prescribing rate. The latter may be explained by the low baseline prescribing rate in Switzerland for pediatric LRTI and the choice of an inappropriately low PCT cut-off level for this population.

**Trial Registration:**

Controlled-Trials.com ISRCTN17057980 ISRCTN17057980

## Introduction

Lower respiratory tract infection (LRTI) is a leading cause of morbidity and mortality in children and adolescents worldwide; pneumonia is the number one cause of childhood mortality worldwide, and in Europe accounts for 9% of deaths in children under 5 years of age. Depending on age and diagnostic methodology, a bacterial etiology has been shown to occur in 33 – 70% of pneumonia in children. The lack of clinical, radiological, and laboratory tests to safely rule out bacterial involvement in LRTI still drives antibiotic treatment today. A reduction of antibiotic exposure in children with LRTI could be expected to have an impact on antibiotic consumption and the development of antibiotic resistance worldwide [1]–[11].

Procalcitonin (PCT) guided treatment for respiratory tract infections has been shown to markedly reduce antibiotic exposure in adults [12]–[18]. Smaller, single center trials have suggested that PCT may be helpful in the pediatric patient population [19], [20]. The purpose of the ProPAED trial was to investigate whether PCT guided treatment can reduce the antibiotic prescribing rate and the duration of antibiotic treatment in children and adolescents with LRTI presenting to an emergency department using the cut-off ranges successfully established in adults.

## Methods

### Trial design and participants

The protocol for this trial and supporting CONSORT checklist are available as supporting information; see Protocol S1 and Checklist S1, respectively. We included all children and adolescents, 1 month to 18 years of age, presenting with LRTI to the emergency departments of two pediatric hospitals in Switzerland (Basel, Aarau) between 01/2009 and 02/2010 regardless of antibiotic treatment history. Patients were excluded if they or their care-takers were unwilling to participate or were unable to give **written informed consent** due to language problems. Additional exclusion criteria were severe immune suppression (HIV infection with a CD4 count <15% of normal age-specific counts), immunosuppressive treatment, neutropenia (<1000×10^9^/L), cystic fibrosis, acute croup, hospital stay within previous 14 days, or other severe infection.

The trial was approved by both the ethics committee of the University Basel and Kanton Aargau and conducted according to the principles of good clinical practice, and supervised by a steering committee and an independent data safety monitoring board. The trial is registered with the International Standard Randomized Controlled Trial Number (ISRCTN) register (number 17057980).

Acute LRTI was defined as the presence of fever (core body temperature ≥38.0° C measured in hospital or at home) and at least one symptom (cough, sputum production, pleuritic pain, poor feeding) and at least one sign (tachypnea, dyspnoea, wheezing, late inspiratory crackles, bronchial breathing, pleural rub) for less than 14 days. In the case of fever, poor feeding and tachypnea without other signs, persistence of tachypnea following effective antipyretic treatment was required. Community acquired pneumonia (CAP) was defined as acute febrile LRTI with a new or increasing alveolar infiltrate on chest radiograph as assessed by the attending pediatrician. Non-CAP LRTI (i.e. bronchitis, bronchiolitis) was defined as acute febrile LRTI presenting with hyperinflation or new or increased peribronchial infiltrates without alveolar infiltrates on chest radiograph. Fever was considered a necessary sign, because antibiotic treatment for potential bacterial LRTI would not usually be considered in a pediatric patient with an afebrile LRTI.

To assess the potential of recruitment bias, all patients with LRTI seen in the emergency departments of the study hospitals during the trial were identified by retrospective chart review.

### Randomization

Eligible patients were randomly assigned to either PCT guided antibiotic treatment (PCT group) or to clinically guided standard care (control group) by a pre-specified computer-generated scheme (1:1 ratio). Patient allocation was concealed by use of web-based online patient registration. We used variable blockrandomization with stratification for the participating clinic and the type of LRTI.

### Procedures

After obtaining informed consent and performing randomization (day 1), blood samples for PCT, C-Reactive Protein (CRP) and full blood counts (FBC) plus nasopharyngeal aspirates were taken from all participants. Performance of chest X-ray was encouraged for all patients. Serum PCT was measured by B.R.A.H.M.S. PCT sensitive Kryptor® (B.R.A.H.M.S., Hennigsdorf, Germany), a rapid sensitive assay with a functional sensitivity of 0.06 µg/L and a lower detection limit of 0.02 µg/L with an assay time of less than 30 minutes. CRP was measured by an immunoturbidimetric assay, the Tina-Quant C-Reactive Protein Generation 3 assay (Roche Diagnostics, Mannheim, Germany) on Hitachi 912 Modular P analyzer. For this test, functional sensitivity is 0.6 mg/L and detection limit 0.3 mg/L. FBC was done by Sysmex xT-2000i and differentiation was performed manually. On days 3 and 5, patients were re-evaluated clinically and PCT measurements were repeated.

### Antibiotic guidance and endpoint assessment

In the PCT group, initiation, continuation or termination of antibiotic treatment was strictly guided by PCT cut-off levels used in previous trials in adults with LRTI [12], [14], [21]–[23]. The algorithm provides PCT based decision categories for the likelihood of requiring antibiotic treatment for bacterial LRTI: “definitely” (>0.5 µg/L), “probably” (0.26–0.5 µg/L), “probably not” (0.1–0.25 µg/L), and “definitely not” (<0.1 µg/L). The PCT algorithm could be overruled for patients with life threatening infections, defined as severe co-morbidity, emerging ICU need during initial follow-up, or hemodynamic or respiratory instability. For all patients, discontinuation of antibiotics was encouraged upon clinical stabilization and when PCT values fell below 0.25; for patients with initial PCT values >10 μg /L when levels decreased below 90% of the initial value. Continuation of treatment on day 5 was determined according to the following algorithm: >1 μg/L: 7 days, 0.51–1 μg/L: 5 days, 0.26–0.5 μg/L: 3 days, and ≤0.25 μg/L: no antibiotic. In the control group, antibiotic treatment was initiated based on physician assessment and clinical guidelines for a duration of 7–10 days for uncomplicated CAP and 14 or more days for complicated CAP, e.g., parapneumonic effusions, empyema, abscess [24]. The hospital outpatient services and responsible non-hospital based primary care pediatricians were informed about study procedures and given guidance concerning assessment of adverse events.

Children 14 years of age or older, or care takers of children under 14 years of age, completed a diary from day 1 through 14 including items on antibiotic intake, consumption of other medication, hospitalization, and occurrence of standardized symptoms. A questionnaire and visual analogue scale (0 to 100%) for self-assessment of impairment of overall daily activity thought attributable to the LRTI was also distributed [25], [26]. For endpoint assessment each patient was contacted on day 14 by a study pediatrician blinded to treatment allocation of the child. Contact consisted of a structured telephone interview with the parents.

Safety monitoring included assessment of complications of LRTI: occurrence of serious adverse events (SAE) or disease specific failure including hospital readmission, recurrent infection requiring antibiotics, any co-morbidity in need of antibiotics, or worsening of impairment of daily activity by ≥20% on the visual analogue scale according to parent interview and diary.

### Statistical analyses

The primary endpoint of this trial was antibiotic prescibing rate within 14 days of randomization; secondary endpoints were (i) duration of antibiotic treatment, (ii) rate and duration of side effects of antibiotic treatment, (iii) rate and duration of hospitalization, (iv) occurrence of serious adverse events, complications of LRTI or disease specific failure, and (v) impairment of daily activity attributable to LRTI during the 14 days following randomization.

For sample size calculation, we assumed that PCT guidance would reduce antibiotic prescribing from 90% to 60% and from 30% to 15% in children and adolescents with CAP and non-CAP LRTI, respectively. With a 2-sided type I error rate of α = 0.05, 64 and 242 patients with CAP and non-CAP LRTI, respectively, had to be included to attain a targeted power of 80%. Assuming that 20% of all randomized patients would have CAP, a total sample size of 320 patients was determined, giving a power of 93% to detect a decrease in antibiotic prescribing from 42% (control group) to 24% (PCT group) for all LRTI patients.

In an intention-to-treat analysis, we used a two-sided chi-squared test to compare the primary endpoint (antibiotic prescribing within 14 days of randomization) between PCT and control groups. We performed this test in all LRTI patients and in the pre-specified subgroups of patients with CAP and non-CAP LRTI according to diagnosis at randomization.

To compare the primary and secondary binary endpoints between PCT and control groups, we estimated the rate difference and the odds ratio by logistic regression. In this model, we additionally included an interaction term between the therapeutic group and diagnosis at randomization (CAP versus non-CAP LRTI) to obtain effect estimates of PCT guidance in the two pre-specified subgroups and to investigate differences in effect of PCT guidance between patients with CAP and non-CAP LRTI. For secondary continuous endpoints, we used the Wilcoxon rank sum test and report the estimated mean difference between PCT and control group. We used exploratory statistics to assess mean impairment of daily activity. Confidence intervals for rate differences were calculated using Newcombe’s method [27], and for mean differences, using the bootstrap percentile method [28]. For our analyses and graphics, we used R version 2.14.0 (R Foundation for Statistical Computing, Vienna, Austria) and the R add-on packages *epiR* version 0.9–32 and *boot* version 1.3–3.

## Results

**Figure 1:**
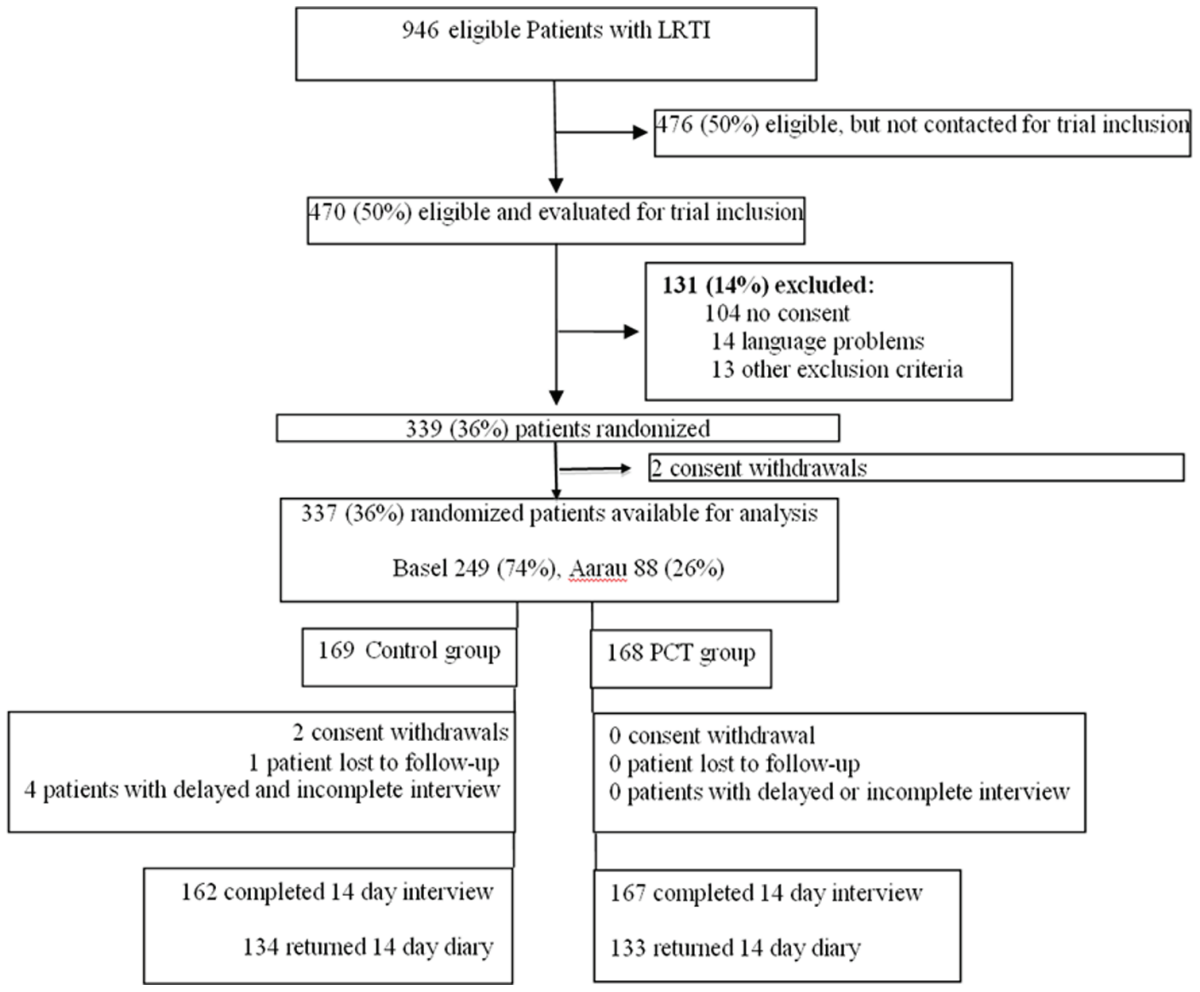
Trial profile.

Of 946 patients with LRTI, 470 were formally screened for eligibility and of those, 337 randomized patients were available for analysis (Figure 1). All eligible patients were formally screened for baseline characteristics and included patients did not differ from excluded patients. However, the study population was more likely to have CAP compared to the population not assessed for eligibility (Table S1). Follow-up was complete for 329 (98%) patients with a telephone interview after 14 days (median 14 days; interquartile range [IQR] 13–15). Clinical recovery could be confirmed for the two patients withdrawing consent and two further patients with incomplete follow-up, all in the control group. We received 208 (62%) complete and 59 (18%) incomplete diaries (median 14 missing values; IQR 10–58). Seventy diaries (21%) were not returned.

Baseline characteristics of randomized patients were similar in both groups (Table 1). Median age in the PCT and control group was 2.7 and 2.9 years respectively; 48% of children in both groups were hospitalized. In 215 (64%) children, the initial diagnosis was CAP.

**Table 1:** Patient baseline characteristics.

	PCT group (N = 168) *		Control group (N = 169) *	
**Demographics**				
Age, years, Median (IQR)	2.7 (1.1–5.2)		2.9 (1.2–5.7)	
Male gender, N (%)	98 (58)		98 (58)	
**Study centre,** N (%)				
Basel	128 (76)		121 (72)	
Aarau	40 (24)		48 (28)	
**Day care,** N (%)				
At home	84 (52)	(N = 162)	86 (52)	(N = 167)
Day care/nursery/school	78 (48)		81 (48)	
**Siblings,** N (%)				
Siblings 0	48 (30)	(N = 158)	39 (23)	(N = 168)
Siblings ≥1	110 (70)		129(77)	
**Vaccination Status,** N. (%)				
*Streptococcus pneumoniae (PCV7)*				
0–2x	115 (74)	(N = 155)	112 (74)	(N = 151)
≥3x	40 (26)		39 (26)	
*Haemophilus influenzae type b*				
0–2x	28 (18)	(N = 157)	27 (17)	(N = 155)
≥3x	129 (82)		128 (83)	
**Clinical history**				
Antibiotic pre-treatment, N (%)	25 (15)		17 (10)	
Days of fever before presentation, Median (IQR)	2 (1–4)	(N = 164)	3 (1–4)	(N = 166)
Fever, N (%)	168 (100)		169 (100)	
Cough, N (%)	167 (99)		169 (100)	
Sputum production, N (%)	62 (37)		79 (47)	
Poor feeding, N (%)	79 (47)		74 (44)	
Pleuritic pain, N (%)	42 (25)		53 (31)	
**Clinical findings**				
Body temperature, °C, Median (IQR)	38.5 (37.9–39.1)	(N = 167)	38.3 (37.8–39.0)	(N = 168)
Respiratory rate, Median (IQR)	40 (30–48)	(N = 156)	40 (28–48)	(N = 164)
Heart rate, Median (IQR)	144 (124–160)	(N = 163)	141 (120–160)	(N = 164)
Tachypnea, N (%)	127 (76)		116 (69)	
Dyspnea, N (%)	110 (65)		107 (63)	
Wheezing, N (%)	53 (32)		48 (28)	
Late inspiratory crackles, N (%)	71 (42)		69 (41)	
Reduced breathing sounds, N (%)	60 (36)		49 (29)	
**Laboratory findings,** Median (IQR)				
PCT, ug/L	0.26 (0.14–1.06)		0.21 (0.12–2.24)	
CRP, mg/L	23 (8–88)	(N = 162)	20 (7–55)	(N = 165)
Leukocyte count, cells/ul	11.9 (8.7–18.9)	(N = 164)	11.3 (7.7–16.4)	(N = 166)
**Diagnosis at randomization,** N (%)				
Non-CAP LRTI	60 (36)		62 (37)	
Community-acquired pneumonia	108 (64)		107 (63)	

Abbreviations: PCT, procalcitonin; IQR, interquartile range; CRP, C-reactive protein; Non-CAP, non-community-acquired pneumonia; LRTI, lower respiratory tract infection. * N in this column indicate the number of individuals with information on a particular variable.

In the PCT group 104 of 168 (62%) patients and in the control group 93 of 165 (56%) patients received antibiotics. The estimated difference in antibiotic prescribing rate between the PCT and the control group was 6% (95% CI −5%, 16%; P = 0.359) in all LRTI patients, 28% (95% CI 12%, 43%; P = 0.002) in the subgroup of 120 patients with non-CAP LRTI and −8% (95% CI −19%, 4%; P = 0.250) in the subgroup of 213 patients with CAP. The odds ratio (OR) of receiving antibiotic treatment within 14 days of randomization in the PCT compared to the control group was 1.26 (95% CI 0.81, 1.95) in all LRTI patients, 4.09 (95% CI 1.80, 9.93) in non-CAP LRTI patients and 0.66 (95% CI 0.35, 1.23) in CAP patients (Tables 2 and 3). The interaction term between therapeutic group (PCT versus control) and diagnosis at randomization (CAP versus non-CAP LRTI) indicated a statistically significant difference in the effect of PCT guidance on antibiotic prescribing rate between CAP and non-CAP LRTI patients (OR for interaction 0.16; 95% CI 0.06, 0.45).

**Table 2:** Efficacy and safety, primary and secondary endpoints.

Outcome	Measure	PCT group (N = 168)		Control group (N = 169)		Rate difference, % (95% CI)	Odds ratio (95% CI)	Mean difference (95% CI)
**Primary endpoint**			*		*			
Antibiotic prescription within 14 days of randomization	N (%)	104 (62)		93 (56)	(N = 165)	6 (−5, 16)	1.26 (0.81, 1.95)	
**Secondary endpoints**								
Duration of antibiotic treatment, days	Mean (median [IQR])	4.5 (4 [0–8])	(N = 167)	6.3 (6 [0–11])	(N = 164)			−1.8 (−3.1, −0.5)
Antibiotic side effects*	N (%)	56 (39)	(N = 144)	57 (38)	(N = 149)	1 (−10, 12)	1.03 (0.64, 1.65)	
Duration of antibiotic side effects, days	Mean (median [IQR])	1.4 (0 [0–2])	(N = 144)	1.3 (0 [0–1])	(N = 149)			0.1 (–0.4, 0.7)
Hospitalization	N (%)	104 (62)		100 (60)	(N = 167)	2 (−8, 12)	1.09 (0.70, 1.69)	
Duration of hospitalization, days	Mean (median [IQR])	2.6 (2 [0–4])	(N = 167)	2.7 (2 [0–5])	(N = 164)			−0.1 (−0.8, 0.5)
Safety†	N (%)	38 (23)		33 (20)	(N = 164)	2 (−6, 11)	1.16 (0.69, 1.97)	

* On days of antibiotic therapy patients showing an exanthema or vomiting or diarrhea as stated in the patient’s diary from day 1 up to day 14.

† Occurrence of any of the following entities: **complications** from pneumonia or other LRTI (e.g., parapneumonic effusions in need of puncture, empyema, lung abscess, necrotizing pneumonitis, acute respiratory distress syndrome) or occurrence of **SAEs** (hospital readmission, admission to intensive care unit, unexpected life threatening condition, condition of compromising sequelae or death occurring in the 14 days following the inclusion of the patient) or **disease specific failure**, including hospital readmission, recurrent infection in need of antibiotics or development of any co-morbid condition in need of antibiotics irrespective of the primary LRTI diagnosis, worsening of ≥20% of daily restrictions from LRTI according to parent interview and diary, new onset of respiratory distress or worsening of pre-existing respiratory distress (i.e., tachypnea, and or dyspnea in spite of β_2_-mimetic treatment) or increasing or new onset of O_2_ requirement or development of global respiratory insufficiency – increasing pCO_2_. * number of individuals with available data for a given endpoint.

**Table 3:** Subgroup analyses.

Outcome	Measure	PCT group		Control group		Rate difference, % (95% CI)	Odds ratio (95% CI)	Mean difference (95% CI)
Non-CAP LRTI		(N = 60)	*	(N = 62)	*			
Antibiotic prescription within 14 days of randomization	N (%)	27 (45)		10 (17)	(N = 60)	28 (12, 43)	4.09 (1.80, 9.93)	
Duration of antibiotic treatment, days	Mean (median [IQR])	2.4 (0 [0–5])	(N = 59)	1.6 (0 [0–0])	(N = 60)			0.8 (−0.5, 2.0)
Antibiotic side effects	N (%)	14 (26)	(N = 54)	6 (10)	(N = 58)	16 (1, 30)	3.03 (1.11, 9.22)	
Duration of antibiotic side effects, days	Mean (median [IQR])	1.0 (0 [0–0.8])	(N = 54)	0.5 (0 [0–0])	(N = 58)			0.5 (−0.2, 1.2)
Hospitalization	N (%)	37 (62)		32 (53)	(N = 60)	8 (−9, 25)	1.41 (0.68, 2.93)	
Duration of hospitalization, days	Mean (median [IQR])	2.5 (2 [0–4])		2.3 (1 [0–5])	(N = 60)			0.3 (−0.8, 1.2)
Safety	N (%)	15 (25)		13 (22)	(N = 60)	3 (−12, 18)	1.21 (0.52, 2.85)	

Community-acquired pneumonia		(N = 108)		(N = 107)				
Antibiotic prescription within 14 days ofrandomization	N (%)	77 (71)		83 (79)	(N = 105)	−8 (−19, 4)	0.66 (0.35, 1.23)	
Duration of antibiotic treatment, days	Mean (median [IQR])	5.7 (5 [0–9])		9.1 (10 [4.5–12.3])	(N = 104)			−3.4 (−4.9, −1.7)
Antibiotic side effects	N (%)	42 (47)	(N = 90)	51 (56)	(N = 91)	−9 (−23, 5)	0.69 (0.38, 1.23)	
Duration of antibiotic side effects, days	Mean (median [IQR])	1.7 (0 [0–2])	(N = 90)	1.8 (1 [0–3])	(N = 91)			−0.1 (−0.9, 0.6)
Hospitalization	N (%)	67 (62)		68 (64)		−2 (−14, 11)	0.94 (0.54, 1.63)	
Duration of hospitalization, days	Mean (median [IQR])	2.6 (2 [0–4])	(N = 107)	2.9 (2 [0–5])	(N = 104)			−0.3 (−1.1, 0.5)
Safety	N (%)	23 (21)		20 (19)	(N = 104)	2 (−9, 13)	1.14 (0.58, 2.24)	

Abbreviations: PCT, procalcitonin; CI, confidence interval; Non-CAP, non-community-acquired pneumonia; LRTI, lower respiratory tract infection. * Number of individuals with available data for a given endpoint.

**Figure 2:**
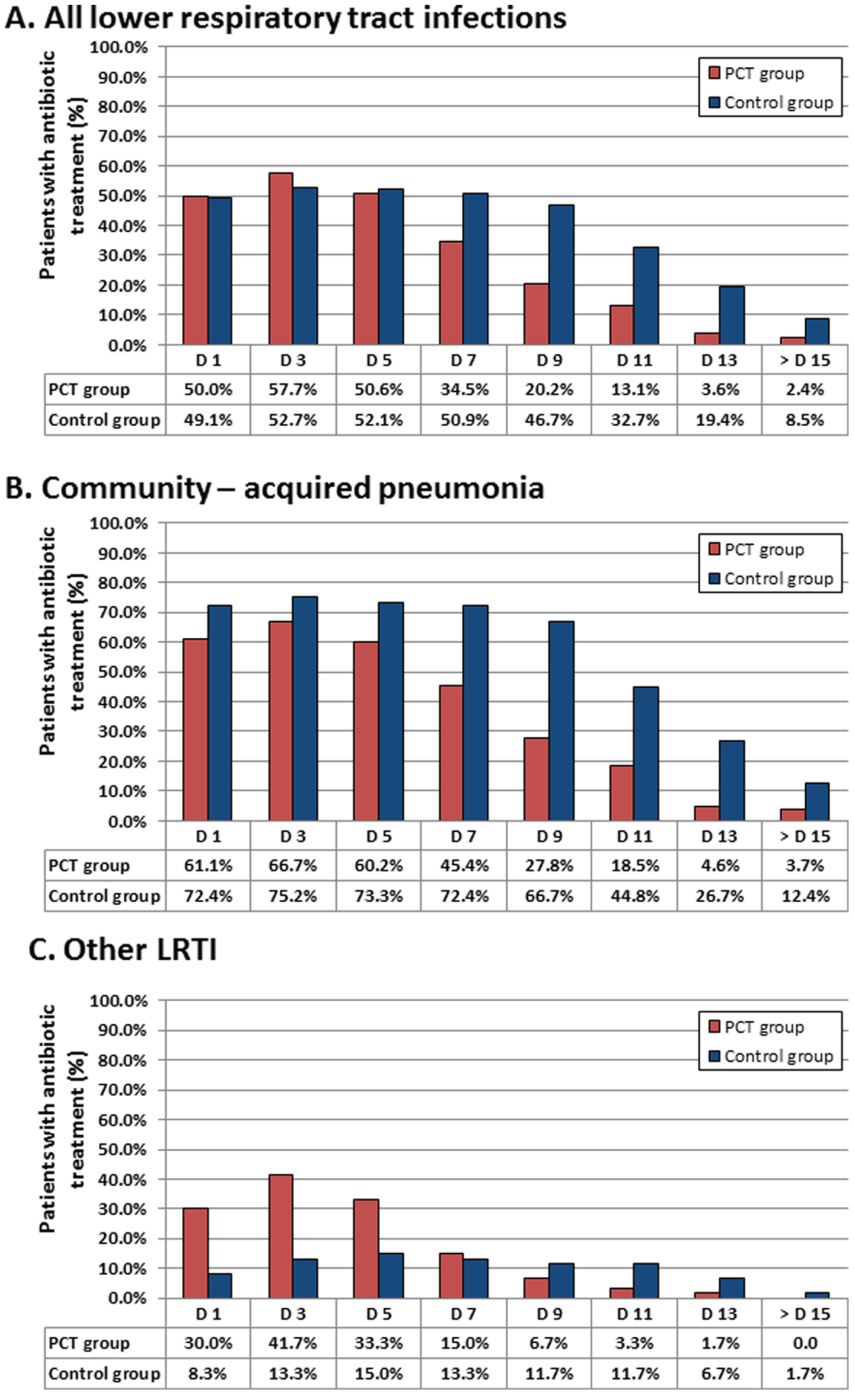
Antibiotic prescribing rate. Antibiotic treatment by day since randomization for all children and adolescents with lower respiratory tract infections (LRTI) and for pre-specified subgroups according to PCT guidance and control. (A) All lower respiratory tract infections; (B) Community-acquired pneumonia (CAP); (C) Bronchitis and Bronchiolitis (non-CAP LRTI).

**Figure 3:**
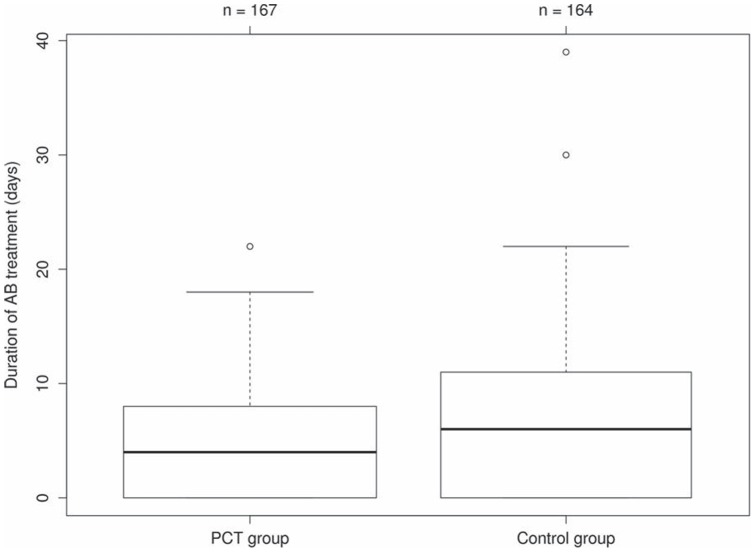
Duration of antibiotic treatment. Box plots of the distribution of the duration of antibiotic (AB) treatment (in days) for children and adolescents with lower respiratory tract infection (LRTI) in the procalcitonin (PCT) and control group.

For each diagnostic group (all LRTI, CAP, non-CAP LRTI), the proportions of patients receiving antibiotics in the PCT group compared to the control group between day 1 and 14 are shown in Figure 2. In comparison with clinical guidelines, PCT guidance reduced the duration of antibiotic treatment in LRTI patients and in the subgroup of CAP patients. The mean duration of antibiotic exposure was 4.5 and 6.3 days in the PCT and control group, respectively (mean difference −1.8 days; 95% CI −3.1, −0.5; P = 0.039) (Table 2 and Figure 3). In the subgroup of patients with non-CAP LRTI, the mean duration of antibiotic treatment was 2.4 and 1.6 days in the PCT and control group, respectively (mean difference 0.8 days; 95% CI −0.5, 2.0; P = 0.01). In patients with CAP, it was 5.7 and 9.1 days in the PCT and control group, respectively (mean difference −3.4 days; 95% CI −4.9, −1.7; P<0.001) (Table 3).

Rates of side effects from antibiotic treatment, hospitalization, and the combined safety endpoint (including SAE, complications of LRTI, and disease specific failure) were similar in both study groups. The rate difference for the combined safety endpoint between PCT and control group was 2% (95% CI −6%, 11%), and the OR was 1.16 (95% CI 0.69, 1.97) (Table 2).

**Figure 4:**
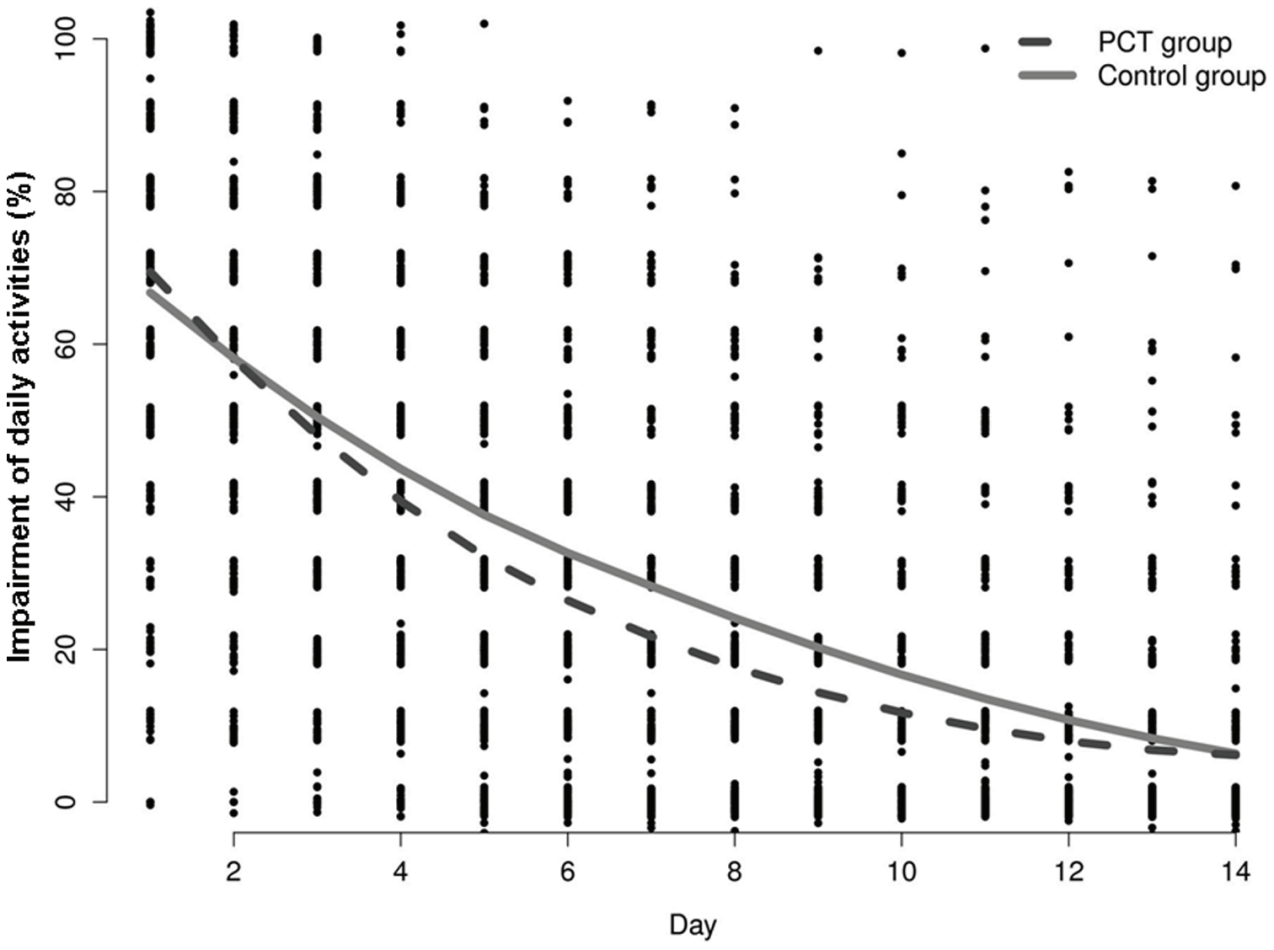
Impairment of daily activities. Impairment of daily activities attributable to lower respiratory tract infection (LRTI) over time in 267 children and adolescents who returned diaries in the procalcitonin (PCT) and control group. The smooth curves are local averages calculated using the default loess smoother in R.

In the subset of 267 patients who returned their diaries, mean impairment of daily activity attributable to LRTI declined during the 14 days following randomization in both PCT and control group patients, indicating no relevant difference between the two study groups (Figure 4).

## Discussion

Compared with clinical guidelines, PCT guidance did not reduce the antibiotic prescribing rate in children and adolescents with LRTI. However, antibiotic treatment duration was reduced.

The failure to reduce the rate of antibiotic prescribing for children with LRTI may be due to several factors. First, pediatricians in Switzerland have a low rate of prescribing antibiotics in general. For example, in a preparatory study for this trial, the background antibiotic prescribing rates for LRTI, CAP, and non-CAP in children and adolescents in the greater metropolitan area of Basel were 72%, 93%, and 19%, respectively (Reppucci R, et al. unpublished data). In the present study antibiotics were prescribed for 79% of CAP and 17% of non-CAP patients in the control group, which is even lower than the 89% background rate. We interpret this observation as a Hawthorn effect introducing bias towards the null hypothesis. Second, the PCT cut-off values we used to guide decision-making on initiating antibiotic treatment, based on an algorithm successfully established in adults with LRTI, may have been too low for use in children with LRTI, especially those with non-CAP LRTI. The effect of low PCT cut-off levels would be more pronounced in patients expected to have low PCT levels close to the cut-off level, such as patients with non-CAP LRTI. In our study, although there was a trend for PCT guidance to reduce antibiotic prescribing in the CAP subgroup, there was an increased rate in the non-CAP LRTI subgroup.

Regardless of subgroup, LRTI patients in the PCT group were treated with antibiotics for a shorter duration than controls. This reduction in antibiotic duration was most pronounced in the sub-group of patients with CAP. This is consistent with findings in adult patients with LRTI [12], [14], [22], [23] and in neonates treated for suspected sepsis [20].

Strengths of our study are the concealed allocation of patients and the excellent follow-up98% of patients79% return rate of diariesThe inclusion rate of CAP was higher than expectedadditional power to the most important clinical patient group. Previous studies suggested that short course antibiotic treatment in children with uncomplicated LRTI may be safe and effective [29]–[31]. Our trial indicates that PCT measurement identifies the children with complicated and uncomplicated LRTI in whom antibiotic treatment can be discontinued early even in the absence of known microbial etiology.

This trial was not powered to assess safety by a non-inferiority design. However, there were favorable outcomes in all patients with no adverse effects attributable to early termination of antibiotic treatment. Although predictive determinants of the appropriate duration of antibiotic treatment for pediatric LRTI were lacking, previous studies suggest that short course antibiotic treatment in children with uncomplicated LRTI may be safe and effective [29]–[31].

PCT guidance in children with LRTI did not reduce the rate and duration of hospitalization. This is most likely due to the fact that determinants for admission were hypoxemia, failure to take oral fluids, or the need for intravenous antibiotic treatment. These factors are independent of PCT levels.

In a recent single center trial in hospitalized children with CAP, which used the same PCT algorithm as in the present study, PCT guidance reduced the antibiotic prescribing rate by 14%, while 100% of patients in the control group received antibiotics (19). PCT guidance in this Italian study reduced antibiotic prescribing rates to levels comparable to our baseline rate in CAP control patients. There may have been several reasons for this. Our study population may have been more severely ill. For example, the mean PCT levels in hospitalized CAP patients in the Italian study were lower (PCT group: 1.8 ug/L; control: 1.8 ug/L) in comparison to the hospitalized CAP patients in our study (PCT group: 4.5 ug/L; control: 6.9 ug/L), in spite of using the same assay for PCT measurements. Also, in our study, all CAP patients had alveolar consolidation as assessed by the emergency care pediatrician, whereas 35 – 39% of patients in the Italian study only showed reticulo-nodular infiltrates based on the *post hoc* chest radiograph assessment of a single radiologist.

In conclusion, our results suggest that PCT guidance of antibiotic treatment in children and adolescents with LRTI is feasible, and can contribute to a reduction in antibiotic exposure overall. Cut-off values derived from trials in adults with LRTI, however, may not be appropriate in pediatric patients with LRTI. Future research should focus on determining optimal PCT cut off values for children with LRTI to identify patients who require antibiotic treatment as well as those in whom antibiotic treatment can be withheld safely. As the baseline complication and mortality rate of pneumonia in Switzerland is low, it would be useful to demonstrate the safety of PCT guided short course treatment in pediatric populations at risk of higher rates of complications and mortality. Reducing antibiotic treatment in pediatric patients through PCT guidance could have an impact on overall antibiotic prescribing, as the burden of viral respiratory tract infections in this population is high, and there is a paucity of reliable tests to guide prudent antibiotic use [32]–[37].

## Supporting Information

Table S1 **Characteristics of included, excluded, and missed patient populations.**
(DOC)

Checklist S1 (DOC)

Protocol S1 (DOC)
