# Supplementary material for: Procalcitonin Guidance to Reduce Antibiotic Treatment of Lower Respiratory Tract Infection in Children and Adolescents (ProPAED): A Randomized Controlled Trial
Source: PLoS One. 2013 Aug 6;8(8):e68419. doi: 10.1371/journal.pone.0068419 (PMC3735552; doi:10.1371/journal.pone.0068419)
Supplement: Table S1 — Characteristics of included, excluded, and missed patient populations. (DOC) [file pone.0068419.s001.doc]

**S3**

**Table S1.** Characteristics of included, excluded, and missed patient populations.

|  | Measure | Patients included  (N=337) |  | Patients excluded (N=131) |  | Missed patients  (N=476) |  |
| --- | --- | --- | --- | --- | --- | --- | --- |
| Age, years | Mean (median [IQR]) | 3.8 (2.8 [1.2-5.3]) |  | 4.04 (2.8 [1.1-5.2]) | (N=128) | 3.3 (2 [1.1-4.2]) |  |
| Antibiotic treatment | N (%) | 197 (59) | (N=333) | 70 (54) | (N=130) | 198 (42) | (N=470) |
| Hospitalization | N (%) | 204 (61) | (N=335) | 63 (48) |  | 209 (44) |  |
| CAP | N (%) | 215 (64) |  | 71 (54) |  | 224 (47) |  |
| ICU | N (%) | 9 (3) |  | 1 (1) | (N=130) | 19 (4) | (N=469) |
| Antibiotic treatment in hospitalized patients | N (%) | 132 (65) | (N=202) | 42 (67) | (N=63) | 126 (60) | (N=209) |
| CAP in hospitalized patients | N (%) | 135 (66) | (N=204) | 42 (67) |  | 142 (68) |  |
