# Supplementary material for: Procalcitonin Guidance to Reduce Antibiotic Treatment of Lower Respiratory Tract Infection in Children and Adolescents (ProPAED): A Randomized Controlled Trial
Source: PLoS One. 2013 Aug 6;8(8):e68419. doi: 10.1371/journal.pone.0068419 (PMC3735552; doi:10.1371/journal.pone.0068419)
Supplement: Protocol S1 — (DOC) [file pone.0068419.s003.doc]

**Effect of procalcitonin guidance**

**on antibiotic use, Quality of care, hospitalization, and time to recovery in children with lower respiratory tract infections**

**The “ProPAED” Study**

The ProPAED-Study Investigators as listed in the Appendix

**Steering Committee:** J. Bonhoeffer^1^ (chair, PI), HC. Bucher^2^, U. Heininger^1^, B. Müller^3^, U.B. Schaad^1^

**Data Safety & Monitoring Board:** J. Hammer^1^ (chair), U. Zumsteg^1^, A. Amacher^1^, G. Berthet^4^

**Endpoint Assessment Committee:** D. Trachsel^1^ (chair), J. Schneider^1^, J. Staehelin^4^

**Co-Investigators:** M. Gambon^1^, D Reppucci^1^, D Trachsel^1^ , M. Buettcher^4^, G. Szinnai^1^.

**Statistical Analysis:** J.Schäfer^2^, P.Schütz^5^

**Webmaster:** Oriented.Net

^1^University Children’s Hospital Basel, Römergasse 8, CH-4058 Basel, Switzerland

^2^ Basel Institute for Clinical Epidemiology and Biostatistics, Hebelstasse 10, CH-4031 Basel, Switzerland

^3^ Cantonal Hospital Aarau, Tellstrasse, CH-5001, Aarau, Switzerland

^4^ Children’s Hospital Aarau, Tellstrasse, CH-5001, Aarau, Switzerland

^5^ University Hospital Basel, Hebelstrasse 32, 4031 Basel

**Participating Hospitals** [local investigators and responsible paediatrician at local site]

- University Children’s Hospital Basel, Switzerland [U. Heininger, J. Bonhoeffer]

- Children’s Hospital Aarau, Switzerland [J. Staehelin/G. Berthet, M. Büttcher ]

**Short Title:** Procalcitonin and lower respiratory tract infection in children

**Trial Registration:** ISRCTN 17057980 ([http://www.controlled-trials.com](http://www.controlled-trials.com/))

Insurance Number:

**Correspondence / Secretary:** Dr. med. Jan Bonhoeffer, jan.bonhoeffer@ukbb.ch

**TABLE OF CONTENTS**

[1. ABSTRACT 3](#__RefHeading___Toc218755095)

[2. RESEARCH PLAN 4](#__RefHeading___Toc218755096)

[*2.1. BACKGROUND* 4](#__RefHeading___Toc218755097)

[*2.2. RESEARCH EXPERIENCE OF THE STEERING COMMITTEE* 5](#__RefHeading___Toc218755098)

[*2.3. DETAILED RESEARCH PLAN* 6](#__RefHeading___Toc218755099)

[A. OBJECTIVES 6](#__RefHeading___Toc218755100)

[B. STUDY DESIGN 8](#__RefHeading___Toc218755101)

[C. PROCEDURES 10](#__RefHeading___Toc218755102)

[D. POTENTIAL RISKS, ADVERSE EVENTS AND MONITORING BOARDS 17](#__RefHeading___Toc218755103)

[E. STATISTICAL ANALYSIS 19](#__RefHeading___Toc218755104)

[F. ANCILLARY PROJECTS 22](#__RefHeading___Toc218755105)

[G. LIMITATIONS 23](#__RefHeading___Toc218755106)

[H. REGULATORY AND LIABILITY CONSIDERATIONS 24](#__RefHeading___Toc218755107)

[I. TRIAL MONITORING 24](#__RefHeading___Toc218755108)

[J. FUNDING AND PUBLICATION 25](#__RefHeading___Toc218755109)

[*2.4. TIMETABLE* 26](#__RefHeading___Toc218755110)

[*2.5. SIGNIFICANCE AND OUTLOOK* 27](#__RefHeading___Toc218755111)

[*2.6. REFERENCES* 28](#__RefHeading___Toc218755113)

[Appendix 1: Patient information and consent form 31](#__RefHeading___Toc218755155)

[Appendix 1: Patient information and consent form 31](#__RefHeading___Toc218755156)

[Appendix 2: Patient Diary 35](#__RefHeading___Toc218755157)

[Appendix 3: CRF 1- Baseline inclusion 40](#__RefHeading___Toc218755158)

[Appendix 4: CRF 2 – Day 3 44](#__RefHeading___Toc218755159)

[Appendix 5 CRF 3 – Day 5 47](#__RefHeading___Toc218755160)

[Appendix 6: CRF 4 –Day 14 50](#__RefHeading___Toc218755161)

[Appendix 7: Patient discharge information sheet 52](#__RefHeading___Toc218755162)

[Appendix 8: Office Paediatrician Information sheet 54](#__RefHeading___Toc218755163)

[Appendix 9: SAE report form to DSMB 56](#__RefHeading___Toc218755164)

[Appendix 10: DSMB decision form 58](#__RefHeading___Toc218755165)

[Appendix 11: Guideline for antibiotic treatment of pneumonia in children 60](#__RefHeading___Toc218755166)

[Appendix 12: Case scenarios of study flow 62](#__RefHeading___Toc218755167)

[Appendix 13: Laborauftragsformular 65](#__RefHeading___Toc218755169)

# 1. ABSTRACT

**Background** Children with acute lower respiratory tract infections (LRTI) are frequently treated with antibiotics (AB) inappropriately and utilize important hospital resources unnecessarily. In adults with LRTI, Procalcitonin (PCT)-guided AB therapy was shown to be a reliable parameter to markedly reduce AB use safely. The present randomized intervention was designed to ascertain the usefulness of PCT guidance in children.

**Aim** To compare an antibiotic treatment strategy based on internationally recognized guidelines versus PCT-guidance in children with LRTI with respect to use of AB, clinical outcome and hospital resources.

**Design** Investigator-initiated, randomized controlled trial with an open intervention.

**Setting** Pediatric teaching hospitals and tertiary care clinics in Switzerland.

**Patients** We will consecutively enrol and include patients 1 month to 18 years of age with acute lower respiratory tract infection (LRTI) based on clinical diagnosis and patients with X-ray confirmed community acquired pneumonia (CAP) (2 pre-specified subgroups). Patients who have been hospitalized within the previous 14 days, patients with immune-suppression, chronic infection, or a terminal condition, and patients lacking informed consent or with insufficient knowledge of German ( in a family member) will be excluded. After a pilot phase to assure feasibility, study recruitment will continue from January 2009 to January 2010.

**Sample Size** The total sample size is 338 children with LRTI. With this sample size, the power is 80% to detect a reduction in AB prescription rates in both subgroups of children with and without CAP at a 5% significance level. This is based on the assumptions that the AB prescription rate is reduced from 90% to 60% for patients with CAP (excluding CAP) and from 30% to 15% for patients with LRTI, that 20-25% of randomized children will have CAP, and loss to follow-up and major protocol violations will be below 5%.

**Intervention** Stratified by centre and type of LRTI, patients will be randomized to management according to internationally recognized guidelines (“guideline group”) versus PCT-guided AB therapy ("PCT group"). In the control group, the use of internationally recognized guidelines for the management of LRTIs will be strictly adhered to. In the PCT group, initiation or continuation of AB will be increasingly discouraged (<0.25 or <0.1 ug/L) or increasingly encouraged (>0.25 or >0.5 ug/L) based on the PCT level. In AB-treated outpatients or discharged patients, the duration of AB will be based on the most recent PCT level. A re-evaluation after 6 to 24 hours is recommended if AB are withheld.

**Endpoints** Primary: Antibiotic prescription rate. Secondary: Time to recovery (days with restriction from LRTI), rate and duration of hospitalization, complication rate, measures of laboratory and clinical outcome, side effects from AB, disease activity scores. Endpoints will be assessed at day 1, 3, and 5 in the form of a medical consultation at the hospital, and after 14 days by structured phone interviews conducted by blinded investigators.

**Working hypothesis** Compared to management according to internationally recognized guidelines, PCT-guidance will lead to reduced AB use overall, earlier detection of LRTI in need of AB treatment and decreased hospitalization rate, with a similar clinical outcome and time to recovery measured by days of restriction due to the LRTI.

**Ancillary projects** Four projects performed alongside to this study will synergize scientific efforts.

**Significance** Respiratory tract infections are the leading cause of medical consultations and antibiotic prescriptions in childhood. Improving the diagnostic accuracy, facilitating early detection of LRTIs in need of AB therapy, reducing AB misuse and decreasing unnecessary utilization of hospital resources for LRTIs will improve patient care and may have a major impact on reducing AB resistance and heath care costs.

# 2. RESEARCH PLAN

## *2.1. BACKGROUND*

Procalcitonin (PCT), a prohormone, is activated through microbial toxins, humoral or cell mediated host response and upregulated in invasive bacterial infections.^1^ Its role in adults as a diagnostic biomarker to guide clinical decision making for antibiotic therapy is well established. ^2-6^ The likelihood of a bacterial infection correlates with increasing levels of circulating procalcitonin (PCT). Clinically relevant cut off ranges to guide initiation and duration of antibiotic treatment have been delineated in adults with community acquired pneumonia (CAP) and sepsis.^7,8^ Intervention trials in adults have demonstrated that PCT-guidance can reduce AB use by almost 50% and shortens duration of therapy by 65% with similar clinical outcomes in patients with mild as well as severe grades of CAP.^9^ While several pediatric studies have evaluated PCT as a biomarker of CAP,^10-18^ evidence is lacking that the findings of PCT guided AB treatment in adults are applicable to children and adolescents.

Respiratory tract infections are the most common cause for consultation of pediatric patients in primary care^19^ and CAP in children remains an important reason for hospital admission. While parapneumonic effusions may occur in 30-50% of cases of CAP, suppurative complications (e.g. empyema, lung abscess, necrotizing pneumonia) occur in only 0.6% of cases in developed countries. The lack of diagnostic tests differentiating LRTI with bacterial from those with viral etiology and the high morbidity and mortality in children with untreated bacterial pneumonia in the pre-antibiotic era still drive antibiotic prescribing today. Thus, the proportion of antibiotic prescriptions per consultation ranges between 30-50% for LRTI and 80-90% for CAP.^20-22^ However, it is estimated that 45 to 70% of LRTI are of viral origin^16, 23-26^. The lack of an accurate clinical case definition of serious bacterial pneumonia limits not only the value of such epidemiologic data and the results of clinical trials studying the appropriateness of AB treatment of LRTI in children, but also clinical decision making. Various biomarkers of infection, including PCT, have been demonstrated to be limited in the differentiation between viral and bacterial etiologies of LRTI. However, the most important clinical performance measure is the differentiation of LRTI in need versus those not in need of AB treatment and this may not be identical with bacterial versus viral etiology, but rather depend on the degree of invasiveness of bacterial infection and the associated inflammatory response. The value of PCT as an additional criterion in the WHO case definition of childhood pneumonia has been demonstrated.^13^ However, a formal assessment of PCT guided therapy in children is lacking in the scientific literature.

The need for antibiotic stewardship in the face of globally increasing antimicrobial resistance has created the clinical dilemma of risking short term morbidity and mortality due to restricted antibiotic use versus risking long term morbidity and mortality as a result of untreatable invasive infections caused by highly resistant organisms. This calls for a careful balance of case and risk management for patients with LRTI. Given the high rate of unnecessary AB use in children with LRTI infections from viral origin there is an urgent need for evidence-based guidance of antibiotic therapy for one of the most prevalent infections in childhood.^21, 27^. However, guidance in the form of training and written guidelines have little effect in promoting prudent use of AB for upper respiratory tract infections. ^28, 29^ In contrast, guidance in the form of diagnostic tests which could identify patients in need of antibiotic therapy by differentiating viral and non-invasive bacterial from invasive bacterial disease in a setting where viral and non-invasive etiologies prevail and is likely to reduce and shorten antibiotic exposure of children. PCT is likely to have a pivotal role in guiding confident restriction of antibiotic use. To establish the promising findings in adults as an international standard of care for children and adolescents, a prospective intervention trial is needed.

LRTIs constitute one of the most frequent causes of hospitalization in children. Data on direct medical care cost (DMCC) for LRTI in children are scarce. However, in Germany, the cost for outpatient treatment of CAP was estimated to be approximately a tenth of the cost of inpatient treatment ^30^. The incidence of hospitalization is inversely proportional to age. This is mainly driven by fluid intolerance as a manifestation of disease. However, switching from intravenous to oral administration of AB is often delayed due to the lack of evidence-based criteria leading to prolonged duration of hospitalization. The current recommendation is a total course of 10 days, despite the recent demonstration in several randomized controlled trials that a shorter course of 3-5 days high dose therapy is safe and sufficient ^31-33^. PCT guided switch from intravenous therapy in favour of outpatient per os therapy or cessation of therapy is thus likely to reduce DMCC significantly and allows families and care takers to return to their regular activities earlier. In an era of cost containment, scientific efforts addressing these important issues are both medically and socio-economically relevant.

## *2.2. RESEARCH EXPERIENCE OF THE STEERING COMMITTEE*

**Jan Bonhoeffer** has authored and co-authored more than 20 original articles and reviews and published his original work in journals including Clinical Infectious Diseases, Infection, Pediatrics, Pediatric Infectious Diseases, and Vaccine, among others (for details see CV & publication list). His main research activities related to immunizations and vaccine preventable diseases. He has considerable methodological experience in study design, coordination of large research networks, data analysis and report writing. Currently, supported by grants from the World Health Organization (WHO) and the European Centres for Disease Prevention and Control (ECDC), he works as a research associate at the Department of Pediatric Infectious Diseases at the University Children’s Hospital, Basel. He was instrumental in the planning phase of this study and will head 2 ancillary projects of the ProPAED study.

**Heiner C. Bucher**, professor and director of the Basel Institute for Clinical Epidemiology and Biostatistics (BICE), University, Hospital Basel is an internist and clinical epidemiologist. Over the past 10 years he has conducted various studies on broad aspects of infectious disease management such as various research projects within the Swiss HIV Cohort Study, clinical trials in primary care, meta-analyses on the effectiveness of antimicrobial agents, and cost- effectiveness analyses. He has lead the BASINUS trial 37, the ARTIME trial (SNF 3200BO-102137)20, 38 and was co-principal investigator in the PARTI trial (SNF 3300C0-107772) and is PI of a new project (Procalcitonin or CRP versus guidelines for patient management of acute RTI in primary care 3200B0-122455/1). He has coordinated an individual patient data meta-analysis of AB vs placebo controlled RCTs in acute rhinosinusitis published in the Lancet (SNF 3200B0-111770). He has authored and co-authored more than 100 original articles in peer reviewed journals and is one of the leading promoters of Evidence-based health care in Switzerland, He has received external funding for the unit that is 100% funded by private funds.

**Ulrich Heininger,** Professor and chair of the Division of Infectious Diseases and Vaccines at the University Children’s Hospital in Basel, Switzerland, since 1998. Previously, he was working in the field of pediatric infectious diseases at the University of Erlangen, Germany. He has conducted several vaccine studies as study coordinator and principal investigator in the recent past. His clinical work is in the fields of general pediatrics and infectious diseases. He is also one of the founding members of „The Brighton Collaboration“, an international collaboration of volunteers aiming at standardization of adverse events following immunization. In Switzerland, he is one of the 7 scientists running the INFOVAC-Ped service, a nationwide information network for vaccine related questions raised by physicians in private practice.Prof. Heininger has been appointed to the German and Swiss National Immunization Recommendation Boards in 2001 and 2004, respectively; further, he has been a board member of the European Paediatric infectious Disease Society (ESPID) from 1999 to 2005. Furthermore, he serves on the editorial board of several journals, including Archives of Disease in Childhood, and has published more than 200 scientific publications in the field of paediatric infectious diseases and vaccines.

**Beat Müller** is Professor of Internal Medicine and Endocrinology and Medical Director at the Teaching Hospital Aarau, Switzerland. He overseas several clinical and experimental research projects focusing on the role and regulation of hormokines during illnesses, especially during inflammation and infections.1-17 He was principal investigator of three intervention trials (ProRESP 18, ProCAP 14, ProCOLD 19) and co-principal investigator of a fourth trial (ProDOC/PARTI) 39 closely related to this proposal. Enrolling >1200 patients, these studies validated his concept of safe and more targeted antibiotic stewardship using PCT as biomarker. He has authored and co-authored more than 100 original articles, editorials, book chapters and reviews in journals including The Lancet, New England Journal of Medicine, British Medical Journal, Clinical Infectious Diseases, and American Journal of Respiratory and Critical Care Medicine, among others (see CV & Publication list).

**Urs B. Schaad,** Chairman/Ordinarius of Pediatrics at the University of Basel, and Medical Director of the University Children’s Hospital Basel, Switzerland is a member of various infectious disease committees, professional organizations, and editorial and advisory boards. Urs B. Schaad has published over 250 articles in his field of interest which includes bacterial meningitis in pediatrics (therapy, prophylaxis, in vitro neutrotoxicity studies), pseudomonal infections in cystic fibrosis (therapy, prevention by active immunization), other bacterial infections (e.g., respiratory tract infections), humoral immune responses, vaccinology and new antimicrobial compounds (macrolides, fluoroquinolones).

## *2.3. DETAILED RESEARCH PLAN*

### A. OBJECTIVES

The objectives of this trial are to evaluate whether a PCT guided diagnostic and therapeutic strategy as compared to management and AB prescription based on internationally recognized guidelines without PCT testing will reduce total AB use, and the rate and duration of hospitalization, without leading to a clinically relevant difference in days with restrictions from LRTI in children and adolescents.

Primary Objective:

1) To compare AB use in children with LRTI (acute bronchitis, bronchiolitis and community acquired pneumonia (CAP)) in the presence or absence of PCT testing. The primary endpoint is the AB prescription rate in children and adolescents with LRTI (see definitions below) within 14 days following randomisation.

Secondary Objectives:

2a) To compare days with restrictions from LRTI between the two arms from day 1-14 following randomization. Restriction due to LRTI will be measured with a validated symptom questionnaire for LRTIs by use of diaries. The instrument has been previously used in children and adolescents.

2b) To compare duration of AB treatment (days) between the two randomization groups.

2c) To compare the time to antibiotic treatment between the two randomization groups.

2d) To compare the rate and duration of hospitalization between the two randomization groups

2e) To compare side-effects from AB treatment (duration and severity), including gastro-intestinal signs and allergic reactions between the two randomization groups

2f) To monitor for safety reasons and to compare any complication or disease specific failure in patients managed with or without PCT decision making for AB prescription within 14 days following randomization (see definitions below).

2g) To develop prediction rules for CAP cases in need of AB treatment based on clinical signs and symptoms, biomarkers and chest radiographs.

Working hypotheses for primary and secondary endpoints:

1. PCT guided antibiotic therapy will lead to a lower AB prescription rate, specifically

- a 30% reduction in the AB prescription rate for CAP (control group 90% vs. PCT group 60%)

- a 50% reduction in the AB prescription rate for non-CAP LRTI (control group 30% vs PCT group15%)

2.a. The number of days with restrictions at day 14 in patients of the PCT-guided group will not be higher to those of the control group (mean of 5 days in both groups). We define freedom from restrictions if parents or care takers report over all restrictions to below the 25^th^ percentile and when reporting no or little problem on the symptom questionnaire.

2b. There will be a shorter duration of AB exposure in the PCT guided group compared to the control group

- 50 % shorter AB duration for CAP (control group 10 days vs PCT group 5 days)
- 25% shorter IV AB duration for hospitalized LRTI (control group 4 days vs. PCT group 3 days)

2c) PCT guided therapy will lead to earlier recognition and institution of AB therapy in LRTI presumed to be bacterial in origin and requiring AB.

2d. Patients with PCT guided AB use will have a 25% shorter hospital stay (4 days versus 3 days) as compared to patients without PCT testing.

2e. Side effects from AB will be similar in both groups.

2f. Complications or disease specific failure rates will be the same in both groups (Of note the trial is not powered to show this. However, an interim safety analysis after 50% of recruited patients is planned).

2g. Traditional biomarkers (C-reactive protein, leucocytosis) and chest radiographs will show limited diagnostic accuracy, i.e. the distinction between bacterial infections requiring antibiotic therapy and non-bacterial infections) as compared to PCT or other biomarkers (see ancillary project II). Novel Biomarkers will aid in the development of a prediction rule for LRTI in need of antibiotic treatment.

### B. STUDY DESIGN

**Study setting**

Emergency and Medical Departments of the following hospitals [target number (N) of recruited patients with LRTI/CAP]

- University Children’s Hospital Basel, Switzerland [160/32]

- Children’s Hospital Aarau, Switzerland [160/32]

**Inclusion criteria**

1. Patients older than 1 month and younger than18 years of age, admitted from the community or a child care facility with an acute LRTI (see definition below), regardless whether antibiotics have already been taken. This includes premature infants (>1mo), asthmatics, and patients with severe developmental delay, provided absence of exclusion criteria.

2. Ability to understand verbal and written instructions and informed consent by parents, family member, or care takers.

**Exclusion criteria**

- Patients or their care taker unable or unwilling to give written informed consent in German.
- Patients or their care takers not understanding German (or other local language) and without a family member able to translate

- Patients with severe immune suppression (e.g. patients infected with human immunodeficiency virus infection and a CD4 count <15% of normal age-specific counts (<12 mo: <0.75 x10^9^/L, 1-5 yrs: 0.5 x10^9^/L, >6 yrs: <200x10^9^/L), patients on immunosuppressive therapy and neutropenic patients (neutrophil count < 1000 x 109/L);

- Patients with cystic fibrosis, acute croup, and those with a hospital stay within 14 days prior to inclusion, an accompanying chronic infection (e.g., osteomyelitis, endocarditis, infections with M. tuberculosis, a deep tissue abscess).

**Definitions of acute LRTI**

Acute lower respiratory tract infection, (LRTI)

Fever (core body temperature >38.0° C measured in hospital or at home) of under 14 days duration

AND

AT LEAST ONE of the following symptoms: cough, sputum production, pleuritic pain, poor feeding

AND

AT LEAST ONE of the following signs on physical examination: tachypnea*, dyspnea (grunting, inter-/sub- or supracostal retractions, prolonged expiratory phase, nasal flaring, bilateral wheeze), late-inspiratory crackles, bronchial breathing, pleural rub.

* The combination: fever, poor feeding and tachypnea is not specific for LRTI. Thus, tachypnea will have to persist following effective antipyretic therapy or any of the other of the above signs will have to be present in addition to fever, poor feeding and tachypnea.

Bronchiolitis:

An infant with

Fever (core body temperature >38.0° C measured in hospital or at home) of under 14 days duration

AND

AT LEAST 2 of the following signs: tachypnea, grunting, inter-/sub- or supracostal retractions, nasal flaring, bilateral wheeze, pre-nasal or inspiratory crackles.

#### OR

hyperinflation on chest x-ray

Acute bronchitis:

Acute LRTI of less than 2 weeks duration

AND

AT LEAST 2 of the following signs: tachypnea, inter-/sub- or supracostal retractions, bilateral wheeze, (e.g., obstructive bronchitis) WITHOUT late inspiratory crackles

OR

new or increased peribronchial infiltrates OR segmental atelectasis WITHOUT alveolar infiltrates on chest x-ray

Community acquired pneumonia (CAP):

Acute LRTI of less than 14 days duration

AND

a new or increased alveolar infiltrate on chest radiograph

**Definition of complications or disease specific failure:**

Complications of CAP:

- Complications from CAP: Parapneumonic effusions in need of puncture, lung abscess, empyema, necrotizing pneumonitis, acute respiratory distress syndrome, recurrent infection in need of AB or hospital readmission.

Disease specific failure:

- New onset of respiratory distress or worsening of pre-existing respiratory distress (i.e., tachypnea, and/or dyspnea (grunting, inter-/sub- or supracostal retractions, nasal flaring), in spite of β_2_-mimetic treatment
- Increasing or new onset O2 requirement or development of global resp insufficiency – i.e. increasing pCO2
- Recurrence of signs of LRTI in patients who had been considered to have recovered
- Need for hospitalisation OR need of antibiotics in patients with acute bronchitis or bronchiolitis untreated between the day of randomisation and the day of physician’s decision to prescribe AB
- Development of any co-morbid condition in need of AB irrespective of the primary LRTI diagnosis (e.g., AOM, meningitis)
- Any life threatening condition in need of hospitalisation, admission to ICU for any reason irrespective of the primary LRTI diagnosis, acute adverse drug reaction, or death.

### C. PROCEDURES

**Monitoring of patient flow**

The patient flow will be monitored according to current guidelines and in agreement with the CONSORT statement^34^. Access to data of all eligible patients with LRTI that are not included into this trial is important. Thus, we will request local investigators to collect baseline data and information on inclusion and exclusion criteria in all eligible patients irrespective whether they are or are not included into the trial. This will allow the comparison of anonymous baseline data of eligible patients who consented to participate with those who did not.

**Baseline data collection**

Baseline data collection in patients will be collected on computer readable case report forms (CRF) Teleform® and contain age, birth date, gender, medical history items (fever, cough, sputum production, pleuritic pain, poor feeding, malaise, current antibiotic medication, vaccination status for *Haemophilus influenzae* and *Str.* *pneumococci*, relevant co-morbidities, allergy or intolerance to AB; questionnaire and visual analogue scale (pre-admittance health state: 1) Previously healthy, 2) Recurrent LRTI.); clinical items (tachypnea, dyspnea (grunting, inter-/sub- or supracostal retractions, prolonged expiratory phase, nasal flaring, bilateral wheeze), inspiratory crackles, bronchial breathing, pleural rub, body temperature, extent of pneumonia (uni-/ multi-lobed pneumonia, pleural effusion); mandatory laboratory tests (PCT in both groups (only in the PCT group will the result be given to the paediatrician); routine lab test in both groups (CRP, leukocytes and blood cell count; imaging studies (chest x-ray - mandatory if CAP is suspected); rationale for hospital admission and duration: Percent contribution of medical (“cure”), nursing (“care”), preference of patients (“comfort”) and urge of relatives (“community”), structural problems of hospital management (“control”), respectively.^35,36^ The sum adds up to 100% and is judged by the attending health care professionals collectively.

**Follow-up data collection**

Primary endpoint:

Rate of AB prescription (from randomisation to day 14). AB prescription rates will be measured as reported by the clinician in charge on the respective case report from and by comparison with charts of hospitalized patients. For discharged patients or those in ambulatory care the relevant information will be gathered from the case report form and through physician contact in case of prescription of any new drug.

Secondary endpoints:

Days with restrictions from LRTI will be measured by the patients/care takers. They will complete a diary consisting of a visual analogue scale (VAS) with a range from 0 to 100 for the overall child’s restrictions from LRTI and a validated symptom questionnaire for LRTIs.^37, 38^ The latter will include fever and 6 symptoms (cough, dyspnea, fluid intake, well-being, sleep disturbance, and activity disturbance). Each of the 6 symptoms will be scored (0=no problem, 1=very little problem, 2=slight problem, 3=moderate problem, 4=bad problem, 5=very bad problem, and 6=as bad as it could be).

The duration of AB treatment will be measured as the number of calendar days on which a given patient received one or more doses of an antibiotic. The time to antibiotic treatment will be measured as the number of hours between randomization and the start of antibiotic treatment. The hospitalization rate will be measured as the number of patients hospitalized in relation to the number of patients included in the respective randomization group. The duration of hospitalization will be measured as the number of calendar days regardless of the time of the day on which the patient was admitted or discharged. Side effects of AB treatment will be recorded according to their duration and severity. They include gastro-intestinal signs or symptoms and allergic type of reactions.

Any complication and disease specific failure (see definition above) has to be reported to the data centre by the clinician within 24 hours and appropriate further investigations including imaging techniques leading to optimal treatment should be initiated. Additional information will be gathered at the phone interview at day 14. Any suspected complication or disease specific failure has to be verified by contacting the paediatrician in charge of the child.

**Informed consent statement**

The study will be approved by the ethics committee of the University of Basel (Ethikkommission beider Basel), the ethic committee of Kanton Aargau and additional local committee in case the study will for recruitment problems be expanded to other clinics. Written informed consent will be obtained from responsible medical directors (“Chefärzte”) at each centre by an investigator protocol agreement. All patients with LRTI included into the study or their care takers have to give written informed consent. Informed consent forms will be only available in German. For enrolment, patients, parents, family members or other care takers must have sufficient knowledge of German to understand the instructions, given by their treating paediatricians and to be able to respond to the phone interview at follow-up.

**Method used to generate the intervention assignment schedule**

Following written informed consent , the paediatrician on duty is required to access the trial’s website and register the patient. The web page will generate an allocation code to either the PCT or control group based on a pre-specified computer-generated randomization scheme. The randomization will be stratified for the participating clinic and the type of LRTI (CAP vs. non-CAP). Randomization procedure will be concealed.

**Method of masking**

This is a randomized open intervention trial. Paediatricians in this trial will know that their AB prescribing behaviour is monitored. Thus, there will be an obvious Hawthorne effect. However, we postulate that PCT guided AB prescription and management will lead to no worse clinical outcome and lead to reduced AB use even in such an artificial setting where paediatricians know that they are monitored for AB prescription. Thus, we believe that this bias will be conservative.

**Outcome assessment and blinding**

Members of the study team in charge of the patients’ telephone interview will be blinded to the randomization of the patients. They will be informed that the purpose of the study is to monitor and evaluate the quality and type of care received by patients with acute LRTI. They will be responsible for collecting data on clinical outcomes and AB use and clinical readmission outside of the study centres on day 14. In case the patient’s parents or care takers are indicating the prescription of any new unnamed drugs following hospital discharge, are unable to give adequate information, or are unavailable for follow-up, the interviewers are obliged to contact the treating paediatrician to obtain missing information. Endpoints will be assessed by an independent endpoint committee of at least 2 clinicians blinded to patient allocation. Endpoint judgment will be based on the case report form and if necessary on anonymised copies of the hospital chart.

If an adverse event is suspected following hospital discharge, the treating paediatrician will be contacted by member of the study team who is not blinded to verify all information and to complete a serious adverse event form within 24 hours following the event for review by the data safety monitoring board (DSMB).

**Data generation, data entry and blinding**

The study staff responsible for data management and data entry will not be blinded in regard to allocation of study participants and the goal of the study. The patient consent form and CRFs baseline form will be sent and faxed immediately to the trial centre following enrolment of the patient. Follow-up forms of day 3 and 5 will be sent immediately to the trial centre. Data will be immediately checked for missing or inconsistent data by the data manager and clinicians will be contacted to compete or correct missing or inconsistent data. Data forms will be electronically scanned and entered into the database by a member of the study centre. We will use optical character recognition software (Teleform®) to scan and enter all data forms.

**Intervention**

Local investigators (e.g. the paediatrician in charge for patient recruitment and inclusion, and the responsible co-investigator from each centre) will receive a structured seminar to become familiar with the details of the protocol, the rationale and the design of the trial and all study forms. They will be introduced to good clinical practice guidelines (GCP) and evidence-based guidelines for the management of patients with CAP. The latter are based on generally accepted guidelines for the treatment of pneumonia in children (AAP, BTS, DGPI) supplemented by additional most recent evidence from relevant clinical trials that have been published since the release of these guidelines. The guidelines have been adapted by infectious disease specialists and have been judged useful and applicable by participating paediatricians. To optimize the implementation of guidelines, for all patients the treating paediatrician will strictly follow web-based guidelines, which are also available as hard copies on the emergency units in participating centres. This will be controlled by email alerts released for every patient screened and recruited, respectively. Antimicrobial agents are prescribed, when possible, according to the resistance pattern of the causative organism. When the causative organism is not known, antimicrobial agents are prescribed according to recent guidelines for empiric antibiotic infections. If the guidelines for antibiotic therapy are overruled, the study centre has to be informed by phone.

**Procalcitonin (PCT) test**

Paediatricians will receive detailed information on how to interpret PCT cut-offs. They will also be informed about results from previous PCT trials. PCT will be measured by using a rapid sensitive assay with a functional assay sensitivity of 0.06ug/L, (Kryptor PCT, Brahms, Hennigsdorf, Germany). The test will be performed at the central lab of each participating Hospital. Calibration of PCT measurements across centres will be performed. The assay time for PCT measurements is less than 20 minutes and results will be routinely available within one hour upon ordering (24 hours a day, 7 days per week).

Re-evaluation of the clinical status and measurement of serum PCT levels will be recommended after 6–24 h in all persistently sick and hospitalized patients in whom AB are withheld. The PCT algorithm can be overruled in patients with immediately life-threatening disease (e.g. patients with severest co-morbidity, emerging ICU need during the initial follow-up, in patients with hemodynamic or respiratory instability.

If the algorithm is overruled and AB are given, an early discontinuation of AB therapy after 3 or 5 days will be more or less endorsed, if PCT levels, checked on day 3 and 5 remain <0.25ug/L. If the PCT-algorithm for antibiotic therapy is overruled, the study centre has to be informed by phone.

A procalcitonin level of **<0.1 ug/L** suggests the absence of bacterial infection and the initiation or continuation of AB will be strongly discouraged. AB therapy can be considered in critically ill patients (i.e. unstable vital signs or ICU admission). If AB are given, an early discontinuation of AB therapy after 1, 3 or 5 days will be endorsed, if PCT levels, remain <0.1ug/L.

A PCT level **between 0.1 and 0.25 ug/L** indicates that bacterial infection is unlikely, and the initiation or continuation of AB will be discouraged. AB therapy can be considered in high-risk patients (i.e. unstable vital signs or ICU admission).

A PCT level **between 0.26 and 0.5 ug/L** will be considered to indicate a possible bacterial infection and the initiation or continuation of AB therapy, respectively, will be encouraged.

A PCT level of **>0.5 ug/L** strongly suggests the presence of bacterial infection and AB treatment and continuation will be strongly encouraged. Persistently elevated PCT levels indicate a complicated course (e.g. empyema, abscess). Conversely, PCT levels may remain relatively low in localized infections.

The same cut-offs will be used for patients pre-treated with AB (i.e. treated with one or more doses of AB prior to admission to the emergency department). PCT levels will be reassessed on days 3 and 5 in patients with ongoing AB therapy, and in case of worsening or delayed recovery of signs and symptoms.

AB will be discontinued using the PCT cut-offs defined above. In all patients with a PCT value >10 μg /L on admission, discontinuation of AB will be encouraged if levels decreased below 80 to 90% of the initial value (e.g. 1 μg /L, instead of 0.25 μg /L). In patients with an initial PCT level >10 μg/L and smaller reductions during follow-up, continuation of AB treatment will be encouraged.

In outpatients or discharged patients in whom uncomplicated resolution of the infection is likely or in patients transferred to an institution not taking part in this trial, the recommended total duration of AB therapy will be based on the last PCT level and will be as follows: >1 μg/L 7 days, 0.51 - 1 μg/L 5 days, 0.26 - 0.5 μg/L 3days, 0.1 - 0.25 μg/L no AB, <0.1 μg/L NO AB!. Parents or care takers will be informed to present to the emergency department, if symptoms persist or increase again.

Together with the hospital discharge letter, the primary care paediatrician of the patient will be provided with a letter informing about the inclusion of the patient into the ProPAED trial and an adverse event form including a contact number of the study centre.

**Figure 1:** Overview of the ProPAED study design with CAP flow diagram

LRTI < 14 days, admitted to the Emergency Department

Baseline data collection and website consultation in all eligible individuals

Patients with LRTI & exclusion criteria

Fulfills inclusion criteria & informed consent

Randomization

Control Group

“Standard Guideline Group”

Intervention Group

“PCT Group”

Initiation of AB based on PCT (μg/L):

<0.1 NO AB! PCT control in 6 to 24 hours

0.1 - 0.25 no AB, PCT control in 6 to 24 hours

0.26 - 0.5 initiate AB

>0.5 INITIATE AB!

Management with initiation of AB treatment and switch from i.v. to po use according to guidelines

**Discharged patients**

Continuation of AB therapy based on last PCT (μg/L):

<0.1 NO AB!

0.1 - 0.25 no AB

0.26 - 0.5 3d AB

>0.5 5d AB

>1 7d AB

If AB withheld: F/U and PCT control 6-24 hours

Follow-up and blood samples on day 3 and 5.

**Outpatients**

Continuation of AB according to guidelines for 7-10 days.

If AB withheld: F/U and PCT control in 6-24 hours

Follow-up and blood samples on day 3 and 5.

**Hospitalized patients**

Continuation of AB based on PCT (μg/L):

< 0.1 NO AB!

0.1 - 0.25 no AB

0.26 - 0.5 start/cont. AB

> 0.5 START/CONT. AB!

If PCT >10 consider stop AB after 80-90% decrease.

If AB withheld: F/U and PCT control in 6-24 hours

Follow-up and blood samples on day 3 and 5.

**Hospitalized patients**

Continuation of AB according to guidelines for 7-10 days.

If AB withheld: F/U and PCT control in 6-24 hours

Follow-up and blood samples on day 3 and 5.

**In hospitalized patients**

Consider discharge, if

- stable vital signs (T<38.5°C <18h)
- NO supplemental O_2_ requirement
- Inhalation interval > 4h
- oral intake is feasible
- adequate gastrointestinal absorption is assumed
- No co-morbidity necessitating further hospitalization

Final clinical assessment and blood sample on the day of discharge

**Day 14 phone interview (primary and secondary endpoint)**

**Management of participants throughout the trial**

Patient selection

Step 1. For all consecutive LRTI patients assigned for routine blood collection on admission a web-based study algorithm will be followed and a blinded baseline data form should be completed by the paediatrician on duty on the admitting unit. A password secured website will provide all study-related information including guidelines and patient flow.

Step 2. The paediatrician checks all inclusion and exclusion criteria.

Step 3. If all inclusion criteria are fulfilled and no exclusion criteria are present the paediatrician explains the trial to the patient/care taker, asks for their participation and obtains informed consent. The decision of the parent/care taker will be respected.

Step 4. If the parents or care taker agree to participate, he or she will be randomized - web-based.

Timing of study procedures

Step 5. Routine blood sampling including collection of 2.5ml of plasma (5 ml blood) will be performed on enrolment and day 3 and 5. This amounts to a maximum of 15 ml of blood over 5 days. This is well acceptable even for infants (see section D, potential risks). Standard laboratory values (e.g. C-reactive protein, white blood cell count) will be determined upon the discretion of the treating paediatrician. These tubes are sent to the laboratory of clinical chemistry at the local hospital. Only for patients randomized to the PCT intervention arm, PCT levels are communicated by the password secured website to the treating paediatrician together with a treatment recommendation for AB based on the PCT algorithm. In the control arm, blood samples for PCT will not be communicated to the treating paediatrician, as the PCT level will be blinded on the website. Should it not be possible to collect 5ml of blood for technical reasons, an approximation will be aimed for and priority will be given to PCT testing (200ul of blood).

In addition routine nasopharyngeal aspirates will be obtained from all patients for detection of respiratory viruses. Samples will be sent to the laboratory of microbiology and processed according to standard practice. Results of patients in both groups will be available to the treating physician according to standard clinical practice.

Step 6. In both groups, hospitalized patients will be reassessed clinically and by blood sampling on admission and day 3 and 5. In patients randomized to the PCT group in whom AB were not initiated, clinical re-assessment and blood sampling will be performed within 6 to 24 hours to exclude a progressive bacterial infection. Details on dosing of all prescribed antimicrobials during the study period will be recorded (i.e. date and time of initial therapy; dose at each dosing change, together with reason for change, date of last dose of each agent, reason for discontinuation, date of resumption of therapy). Intravenous antibiotic therapy should be considered in moderate and severe illness (i.e. unstable vital signs or ICU admission) or when oral intake is not feasible (impaired consciousness or swallowing reflexes, functional or anatomical reasons for malabsorption). In both groups, a switch of AB from i.v. to p.o. should be considered, if patients show stable or improving vital signs and if oral intake is possible.

Step 7. In hospitalized patients, hospital discharge is considered if oral intake is feasible, vital signs are stable > 24h, there is no supplemental oxygen requirement and no evidence of acute serious co-morbidity that necessitates hospitalization. On the day of discharge, all parents/care takers will receive 2 leaflets providing general information for the patient and information for their private office paediatrician about the study purpose, the 14 day interview and advice to seek medical attention immediately in case of worsening symptoms of LRTI or an adverse event.

Step 8. In patients in the PCT group who are treated as outpatients or are discharged early the duration of AB therapy is guided by the last PCT level. In outpatients with a complicated course of disease, a follow-up exam in the emergency room (ER) will be scheduled where again clinical and potentially laboratory investigations are performed (including PCT). In patients in the control group who are treated as outpatients or are discharged early the duration of AB therapy is continued according to guidelines.

Follow-up

Step 9. All patients in both groups will be followed-up by a structured phone interview on day 14 (range 12 to 16). In case of incomplete information or in case a patient confirms having received at least one additional drug or prescription following hospital discharge interviewers are obliged to contact the treating GP and obtain additional information. The interviews will be standardized according to the protocol and the blinded investigators will be instructed during an introductory course. Items will be gathered to complete information needed for calculation of primary and secondary outcomes.

General provisions

Any additional diagnostic testing in patients in both groups is at the full discretion of the treating paediatrician. Final decision for the prescription of AB in patients of both groups is at the discretion of the treating paediatrician. However paediatricians should choose the type of AB in accordance with the guidelines and for patients randomized to the PCT group in accordance with PCT guided decision rules as established in the protocol. Duration of AB treatment for patients in the control group will be according to the guidelines and for patients in the PCT group according to the rules of the protocol.

**Data gathering**

Data from all consecutively recruited patients with LRTI, intended for routine blood collection irrespective of whether they enter the trial or not at either study site will be entered in the baseline data form including all baseline measurements. Study paediatricians will receive training during the introduction to the trial in order to make data gathering as fast and simple as possible. The data forms will be pre-tested for suitability and consistency.

**Data management**

Hard copies of patient information sheets, consent forms, patient questionnaires and case report forms (CRFs) will be available at the emergency department at each site. Patient information sheets will be handed out by the nursing staff of the emergency unit at the time of triage of the patient, to utilize patient waiting time prior to consultation with the treating paediatrician. Following medical consultation, signature of the consent form, completion of the patient questionnaire and CRF 1, these documents will be faxed and sent to the study centre immediately on the day of recruitment. The medical record will be marked with a study label for Source Document Verification. On day 3 and 5 the respective CRF will be completed, by the responsible paediatrician on the ward and sent immediately to the study centre. The structured interview at 14 days will be sent to the study centre on completion of the respective interviews. All forms will be scanned at the study centre by the use of Teleform® and entered into the study data base. The study centre will monitor and promote timely completion of all data forms by use of a log file and email and telephone alerts of the local investigators. The data base will be stored on the central server of the UKBB (with 24 hour back-up service). Access to the database will be password protected and will only be allowed for the data manager and the PI. All forms and the database will be kept and archived by the PI for 10 years.

### D. POTENTIAL RISKS, ADVERSE EVENTS AND MONITORING BOARDS

The study investigators will review the data and study conduction on a weekly basis and determine whether the study should continue unchanged or whether modifications to the protocol and/or consent form are required.

**Potential Risks:**

We consider the risks of this study to be low. The specific risks are as follows: Blood Loss: 5ml of blood will be drawn at day 1, 3 and 5. The maximum total expected blood loss for this study will be 15 ml. With a total blood volume of 85ml/kg body weight, an average newborn will have a blood volume of 240-300ml. The physiological significance of the loss of this amount of blood in a newborn is comparable to less than half the amount of blood (250 ml) an adult would lose when making a standard blood donation. Considering that we do not include newborns in the study (inclusion for study begins at 4 weeks of age), the maximum blood loss in our study population does not constitute a risk, even for the smallest children potentially enrolled.

Delayed or omitted antibiotic treatment: based on adult data, we hypothesize that the use of PCT will 1) expedite intitiation of AB treatment in those patients in whom a bacterial infection is present and in whom other markers have shown a more delayed response and 2) will prevent unnecessary AB treatment of patients in whom a bacterial infection is not present. In both cases an improvement in quality of care is expected. Should nonetheless a patient with a bacterial infection incorrectly be assigned not to receive treatment, close monitoring with very short follow-up times (within 24 hours) will minimize any adverse effects.

It is theoretically conceivable that a patient might suffer complications from infections (e.g., abscesses, sepsis, and death) due to delayed or omitted antibiotic therapy based on a falsely low PCT level. Importantly, in all adult trials the adverse event rate was similar in patients with LRTI assigned to the PCT and the control group. Nevertheless, every marker can have false negatives, including PCT; therefore, the PCT algorithm can be overruled in patients thought to be severely ill or in a life-threatening condition.

**Data Safety Monitoring Board (DSMB)**

The trial will be supervised by an independent data safety monitoring board that will not be directly involved in the design and conduct of the trial, or in recruitment of patients. The board will consist of an intensive care specialist and pulmonologist (J. Hammer, chair), two consultants in outpatient pediatrics (U. Zumsteg and G. Berthet), and a pediatric pulmonologist and primary care paediatrician (A. Amacher). The board will supervise the recruitment of patients, patient flow and patient interviews. The board will have access to the randomization code, if necessary. The board will review any serious adverse event within 7 days and report to the principle investigator with a structured form. It has the authorization to request the principle investigator to stop the trial or it may request modifications of the protocol in relation to any problems or adverse events that may occur during the trial. If this occurs the PI is obliged to advise the ethics committee (EKBB) within 24 hours.

**Adverse events**

An adverse event in a subject is defined as any untoward occurrence of any unfavourable and unintended clinically relevant medical sign, symptom, or disease temporally associated with the study which does not necessarily have a causal relationship with the study procedure. Adverse events will be monitored for every subject participating in the study and attributed to the study procedures / design by the study investigators and the data safety and monitoring board according to the following categories in a structured form:

Highly likely: Adverse event(s) is/are related to investigational procedure(s)/agent(s) or other intervention.

Likely: Adverse event(s) may be related to investigational procedure(s)/agent(s) or other intervention.

Unlikely: Adverse event(s) is/are unlikely to be related to investigational procedure(s)/agent(s) or other intervention or clearly related the patient’s baseline condition or medical complications to this.

Highly unlikely: Adverse event(s) is/are not related to investigational procedure(s)/agent(s) or other intervention.

The following grades will be used in defining the severity of adverse events:

Serious: A serious adverse event is defined as the occurrence of readmission, admission to intensive care unit, unexpected life threatening condition, condition with compromising sequelae, or death occurring in the 14 days following the inclusion of the patient. Any serious adverse event has to be reported by fax to the study centre within 24 hours. The DSMB and ethical committee will be informed by email monthly with a summary, outline of the cases and the relevant documents (Discharge letter, study forms, if requested).

Non-serious: Mild or moderate adverse event or within normal limits. Non-serious unanticipated adverse events will be reported to the DSBM and ethical committee as part of the monthly reports.

All adverse events must be followed until resolution, until the condition stabilizes, until the event is otherwise explained, or the subject is lost to follow-up. The investigator is responsible to ensure that follow-up includes any supplemental investigations that may be indicated to completely evaluate the nature and/or causality of the AE. This may include additional laboratory tests or investigations, histo-pathological examinations, or consultation with other health care professionals, and reports.

The study centre may request that the investigator perform or arrange the conduct of supplemental measurements and/or evaluations. If a subject dies during participation in the trial, the study centre has to be provided with a copy of any post-mortem findings, including histo-pathology.

**Endpoint Assessment Committee (EAC)**

Endpoints will be assessed independently by members of a board that will not be directly involved in recruitment of patients and will be blinded towards the allocation of the patients. The board will consist of a pulmonologist and intensive care paediatrician (D Trachsel, chair), a pediatric radiologist (J. Schneider), and two paediatric infectious disease specialists (U. Heininger and J. Staehelin). Two members will review each case, in case of disagreement a decisive assessment by a third member is required.

### E. STATISTICAL ANALYSIS

**Design**

Allocation of patients to either intervention in this randomized controlled trial will be concealed due to the centralized randomization procedure. The randomisation code will be generated by an independent biostatistician not involved in data analysis of the trial. Generator and executor of randomization will be separated. Randomization between the two arms will be 1:1 with variable block sizes of 4-6 and will be stratified by centre (the hospital) and type of LRTI (CAP vs. non CAP).

**Analysis**

Analysis tools

All relevant clinical and laboratory parameters obtained by interview, clinical tests and reviewing of the medical records will be entered by use of Teleform® into an Excel® database. *SAS* (SAS® Institute, Cary, NC, USA), *R for Windows* (R Foundation for Statistical Computing, www.r-project.org) and others will be used for data analysis.

Analysis populations

The primary analysis population is the full analysis set which includes all randomized patients. Patients will be analyzed according to their randomized arm regardless of actual treatment following the intention-to-treat principle. The secondary endpoint 2a (days with restrictions from LRTI) will additionally be analyzed on the per-protocol population which excludes major protocol violators. Specifically, the following patients will be excluded from the per-protocol population for the following reasons: violation of inclusion or exclusion criteria, not treated according to randomized arm (i.e. overriding of PCT algorithm without pre-specified reason), patients lost to follow up. Every effort will be made to keep the number of losses to follow-up minimal.

Sample size considerations for primary endpoint

The primary endpoint of this trial is antibiotic prescription rate within 5 days following randomization. We aim to have sufficient power for testing this endpoint not only in the full population, but also in the subgroups of children with and without CAP separately. Based on results from previous studies^21, 22, 39-41^, we hypothesize that antibiotic-guidance with PCT can reduce the antibiotics prescription rate from 90% to 60% in children with CAP and from 30% to 15% in children without CAP. Based on these assumptions, we would need a sample size of 64 children with CAP and 242 children without CAP to have a power of 80% with a two-sided 5% level of significance in both subgroups. Figure 1 displays power contours with these sample sizes and illustrates what happens if actual prescription rates differ from our assumptions; as an example, if the antibiotic prescription rate in children without CAP were 40% in the control arm instead, the prescription rate in the PCT arm would have to be 23% or lower to be detectable with a power of 80% or more (dashed line in the left plot of Figure 1).

**Figure 1:** Power contours assuming 64 and 242 randomized children with and without CAP, respectively. The plot displays combinations of true antibiotics prescription rates in the two arms that guarantee a power of 80% or 90%, respectively (assuming a two-sided test with significance level 5%). As an example, with 242 children without CAP, power is 80% to show a reduction of antibiotics prescription rate from 30% to 15% or from 40% to 23%.

We assume 20%-25% of all randomized children to have CAP. Therefore, 320 (=64/0.2) evaluable patients are required. This ensures sufficient patients in both subgroups as described above. In addition, with 320 patients, we would have a power of 93% to prove an antibiotics reduction rate in the overall population from 42% to 24%, i.e. the overall prescription rates we expect assuming that 20% of patients have CAP and the prescription rates in the CAP and non-CAP subgroups are as specified above.

We assume approximately 5% of patients to be excluded from the per-protocol analysis. To guarantee sufficient power not only in the primary full analysis set containing all randomized patients but also in the per-protocol population, a total of **338 patients will be randomized.**

Analysis of the primary endpoint

Antibiotics prescription rate during the total follow-up of 14 days following randomization will be compared between the two arms with the χ^2^ test at the 5% significance level. The test will be performed in all patients as well as for patients with CAP and non-CAP separately. In addition, two-sided 95% confidence intervals of rate differences between the two arms will be calculated according to the method of Agresti and Caffo. Patients who drop out before day 5 will be analyzed in the full analysis set according to whether they received antibiotics by the last assessment date or not.

Sample size considerations for secondary endpoints

We only provide power considerations for the secondary endpoint 2a (days with restrictions until day 14 following randomization) for which we aim to show that it is similar in both arms. Under the hypothesis that there is no difference in this endpoint between the two arms, with 338 patients we will have a power of 99%, 86%, and 63%, to exclude differences of 0.5, 0.33, and 0.25 standard deviations(or more), respectively, in favour of the control arm. For example, if the primary endpoint has a standard deviation of 3 days, the chance is 86% that the two-sided 95% t-test confidence interval for the difference between the two arms excludes differences of 1 days or more in favour of the control arm.

Analysis of secondary endpoints

To assess similarity in the days with restrictions until day 14 following randomization between the two arms, a two-sided 95% confidence interval for the mean difference will be calculated based on the t-test unless data suggests strong deviations from normality of this endpoint. In this case, a confidence interval for the difference in location will be calculated based on the Wilcoxon rank sum test instead. All other secondary endpoints will be compared between the two arms by the Wilcoxon rank sum test for continuous data, chi-square tests for categorical data and the log-rank test for time-to-event data. Additional exploratory analyses of endpoints adjusted for important clinical covariates present at baseline will be performed using linear and logistic regression as well as Cox proportional hazards models (as appropriate).

Safety, interim monitoring and analyses

An independent data safety and monitoring board (DSMB) will oversee the trial as described in Section D of the protocol. In addition to the regular monitoring of severe adverse events, a safety interim analysis with regard to complication and disease specific failure rate will be performed after 50% of the patients have been recruited. At this time, the independent monitoring board will be provided with data from this analysis. Based on these data, the committee has to make one of the following recommendations:

- Continue the trial without modification

- Continue the trial with modification

- Stop the trial due to safety concerns (if safe continuation with a modified protocol is not feasible)

The DSMB recommendation should not be based purely on statistical tables but also requires clinical judgment. As the dissemination of preliminary summary data could influence the further conduct of the trial and introduce bias, access to interim data and results will be confidential and strictly limited to the involved statistician and the DSMB and results (except for the recommendation) will not be communicated to the outside and/or clinical investigators involved in the trial.

### F. ANCILLARY PROJECTS

**I) Cost-Effectiveness (Bonhoeffer, et al)**

The comparative analysis of the two groups of the ProPAED study will be conducted from a health care perspective and include cost of all diagnostic procedures, including PCT and other laboratory analyses, therapeutic procedures (e.g., AB use), ‘baseline hospitalization’ costs, doctors’ and nurses’ time, valued according to their wage rates, including time and efforts to enforce PCT algorithm and guidelines, respectively. We will consider direct medical costs including direct non-medical costs and indirect costs derived from leaves or deaths. Periods prior to the study will be compared for endpoints and costs. Cost data will be, collected using micro-costing, i.e. the most detailed approach feasible to costing will be taken. For an analysis from the health care perspective, a wider set of indirect and time costs is considered using the EuroQol questionnaire, adapted to a setting of patients with severe infections. We will define each unit of effectiveness as each patient successfully recovered from CAP, expressed as (QALYs). For the analysis, the incremental cost-effectiveness ratio will be calculated in the study that allows to know which therapeutic option is most effective, i.e., better clinical results with smaller associated costs. Crude cost calculations, sensitivity (assuming different costs for PCT, AB and hospital care) as well as bootstrapping analyses will be performed. Its depth will depend on the available additional funding which will be sought by a different grant application.

**II) Other Biomarkers as diagnostic tools in CAP (Bonhoeffer, et al)**

Both plasma and serum will be routinely asserved and stored in -70°C. Novel biomarkers (e.g., adrenomedullin, IL-6) will be measured as a batch analysis. In patients with CAP, the diagnostic accuracy of standard laboratory tests (i.e. C-reactive protein, white blood cell count,) will be compared to novel biomarkers shown to be promising in adult studies. The utility of these biomarkers in the differential diagnosis of LRTI will be compared. The added value of chest x-rays following diagnoses of LRTI subgroups based on PCT and other novel biomarkers will be evaluated. According to current practice, chest x-rays are obtained in patients with LRTI to identify new or increasing alveolar infiltrates, promting antibiotic treatment of CAP. Based on the results of PCT guided treatment in adults we hypothesise that chest x-rays have limited added value for identifying LRTI requiring antibiotic treatment. Thus the number of chest x-rays (i.e. exposure to radiation) of children with LRTI may be markedly reduced, if treatment of LRTI in children can be guided by PCT and other novel biomarkers.

**III) Identification of novel respiratory viruses (Heininger U, et al)**

CAP is typically diagnosed as a complication of a preceeding URTI. Children presenting to the University Children’s Hospital Basel with CAP are routinely tested for common respiratory viruses (Influenza-, Parainfluenza-, Adeno- and Respiratory Syncythial Virus), and M. pneumoniae. In previous studies we have investigated the clinical and epidemiologic characteristics of human Metapneumovirus in our population [unpublished]. Human boca virus is a novel parainfluenza virus infecting humans and its clinical characteristics are largely unknown. We will aim to identify this and other organism my multiplex PCR in nasopharyngeal aspirates and explore its clinical characteristics. Demonstration of viral pathogens - and associated bacterial superinfections (e.g. S. aureus pneumonia and influenza) - facilitates clinical decision making including optimization of antibiotic regimens and antibiotic step-down therapy.

**IV) Other Novel Methods for Rapid Diagnosis of Bloodstream Infections. (Bonhoeffer J, et al)**

Earlier detection and identification of bacterial microorganisms in blood cultures is essential for improved diagnosis and choice of antimicrobial treatment of patients with bloodstream infections. Growth of microorganisms in blood culture occurs in approximately 5-10% after a median of 24-48 hours depending on the type and quantity of microorganisms. A novel multiplex PCR assay will be applied to optimize further characterization of pathogens identified. We anticipate an improvement in sensitivity (number of positive blood culture) of 10% with an improvement of the detection time of 100% (time until positivity of blood culture between 12-24h) in CAPs. The results of this observation will not influence any treatment decision in patients of the ProPAED study.

### G. LIMITATIONS

**Study feasibility and implications for paediatrician and patient recruitment**

The study will impose additional paper work on treating paediatricians. Therefore, pilot testing of data forms, optimal design and lay-out of data forms that best fit the clinicians need are crucial. Based on our extensive experience from previous studies in adults we are convinced that the trial is feasible. Given the high incidence of LRTI the recruitment period seems realistic and should be sufficient to include the number of required patients per study centre.

**Etiology of CAP**

A state-of-the-art microbiological evaluation is desirable for decisions about appropriate step-down therapy and the use of targeted AB. However, bacteria are identifiable in 10- 60% of paediatric CAP-cases only^16^. This is due to the combined effect of lack of sputum production in young children and the limited sensitivity of blood cultures. In two ancillary projects, we will aim to improve identification and characterization of pathogens associated with CAP.

**Contamination Bias, External Validity and Generalizability**

This is an open “real-life” intervention trial and contamination within the proposed study design is obvious. We expect that in the setting of the study, the AB prescription will be lower as compared to the real-life setting (Hawthorne effect). Similarly, the adherence to the guidelines in the control group may be higher as compared to the real-life setting. However, paediatricians may learn from their experience with PCT testing and change their clinical practice for the treatment of control group patients. For example they might reduce AB treatment or treatment duration in control group patients (spill-over effect). Thus experience gained from treating patients according to the PCT algorithm in the intervention arm or other factors attributable to the conduct of the study (for example increased awareness by paediatricians because of more conscious decision making) may affect AB prescribing in the control arm and could lead to reduced prescribing or treatment duration in the control group. These biases may influence primary and secondary endpoints and cannot be excluded formally, but if we find a difference between the groups the bias is conservative.

The participating hospitals may not be representative and have strong interests or awareness for reducing AB use and this may not be representative for Swiss hospitals in general. As the setting of an emergency room is very comparable in different hospitals in Switzerland generalizability is possible and we consider the external validity to be reasonable. However, we postulate that PCT guided AB prescription management will lead to a reduced AB use even in such an artificial setting where paediatricians know that they are monitored for AB prescription. Thus, we therefore believe that the bias is conservative and generalizability is possible to other settings and hospitals where AB are prescribed more deliberately.

**Confidentiality**

The name of the patients will be known to the data centre and the DSMB as needed. This information will be used only for the purpose of the interviews and otherwise kept strictly confidential. All data forms will be handled as confidential information. The investigator and other trial site personnel will keep any confidential information provided by the coordinating centre related to this trial (including this protocol) and all data and records generated in the course of conducting the trial, and will not use information, data, or records for any purpose other than conducting the trial including ancillary projects. These restrictions do not apply to: (1) information which becomes publicly available through no fault of the investigator or trial site personnel; (2) information which is necessary to disclose in confidence to an institutional ethical committee solely for the evaluation of the trial; or (3) information which is necessary to disclose in order to provide appropriate medical care to a trial subject.

### H. REGULATORY AND LIABILITY CONSIDERATIONS

**Regulatory Considerations**

This study will be conducted in accordance with the most recent version of the Declaration of Helsinki or the applicable International Conference on Harmonization (ICH) guidelines on good clinical practice (GCP) for pharmaceutical studies, adapted with issues relevant to diagnostic studies, whichever represents the greater protection of the individual. The study will be submitted to the institutional review boards of the two involved hospitals and is registered in the Current Controlled Trials Database as “ProPAED”-Study (http://www.controlled-trials.com/ISRCTN). Written informed consent will be obtained from all included patients or their legal representatives. This is an investigator-initiated and -driven study. The sponsor of the study is the University Children’s Hospital Basel, which as such covers insurance costs for liability claims that are related to the fact that an individual was included into this trial and had suffered a complication that is related to the trial. Liability for the technical reliability of the PCT measurement is with the producer of the test (Kryptor PCT, Brahms, Hennigsdorf, Germany). The use of this test and the proposed cut-offs have been validated by four preceding randomized intervention studies including a total of >1200 adult patients. Comparable cut-offs have been used in epidemiological studies in children. Final responsibility for any decision in regard to the use of diagnostic means in addition to PCT and final decision of AB treatment or any other treatment judged necessary for all patients included into this study is with the participating paediatrician. Every participating hospital (“study centre”), represented by the Head Paediatrician (“Chefarzt”) of Paediatrics confirms that he or she keeps himself or herself and all staff employed sufficiently insured against any claims resulting from negligence or malpractice of patients included into the trial.

### I. TRIAL MONITORING

Monitors will periodically contact the site, including on-site visits. The extent, nature and frequency of onsite visits will be based on the enrolment rate, the quality of the documents provided by the site, and consistency of follow-up of the patients according to this protocol.

During these contacts, the monitor will check and assess the progress of the trial, review trial data collected, conduct Source Document Verification and identify any issues and address their resolution. This will be done in order to verify that the data are authentic, accurate, and complete, that safety and rights of subjects are being protected and that the trial is conducted in accordance with the currently approved protocol (and any amendments), GCP, and all applicable regulatory requirements. The investigator agrees to allow the monitor direct access to all relevant documents and to allocate his/her time and the time of his/her staff to the monitor to discuss findings and any relevant issues. In addition to contacts during the trial, the monitor will also contact the site prior to the start of the trial to discuss the protocol and data collection procedures with site personnel.

Quality Assurance

At its discretion, the ProPAED steering committee may conduct a quality assurance audit of this trial. If such an audit occurs, the investigator agrees to allow the auditor direct access to all relevant documents and to allocate his/her time and the time of his/her staff to the auditor to discuss findings and any relevant issues. In addition, regulatory agencies may conduct a regulatory inspection of this trial. If such an inspection occurs, the investigator agrees to allow the inspector direct access to all relevant documents and to allocate his/her time and the time of his/her staff to the inspector to discuss findings and any relevant issues.

Trial and Site Closure

Upon completion of the trial, the following activities, when applicable, must be conducted by the monitor in conjunction with the investigator, as appropriate, namely, return of all trial data to the ProPAED coordinating centre, data clarifications and/or resolutions, review of site trial records for completeness, shipment of stored samples to assay laboratory. In addition, the steering committee reserves the right to temporarily suspend or prematurely discontinue this trial either at a single site or at all sites at any time and for any reason. If such action is taken, selected members of the ProPAED steering committee will discuss this with the respective investigators (including the reasons for taking such action) at that time. The ProPAED steering committee will promptly inform all other investigators conducting the trial if the trial is suspended or terminated for safety reasons. The investigators will inform their local/regional/national regulatory authorities (as appropriate) of the suspension or termination of the trial and the reason(s) for the action. If required by applicable regulations, the investigator must inform the institutional ethical committee promptly and provide the reason for the suspension or termination. If the trial is prematurely discontinued, all trial data must be returned to the ProPAED coordinating centre.

**Records Retention**

In accordance with applicable regulatory requirements, following closure of the trial, the investigator will maintain a copy of all site trial records in a safe and secure location for 10 years. The ProPAED coordinating centre will inform the investigator of the time period for retaining these records in order to comply with applicable regulatory requirements.

### J. FUNDING AND PUBLICATION

Funding

This is an investigator-initiated and -driven study and only very limited funding is available. The study will substantially reduce overall antibiotic use and is *per se* unattractive for pharmaceutical sponsoring. The successful completion of previous trials was dependent on a dedicated study team that was willing to take extra responsibility and work-load in supervising and assuring the rigorous safety-requirements. Since resources to finance overheads were not available, these studies had to operate within the existing framework of local emergency and staff personnel. This pragmatic study design was well received and facilitated a swift implementation into, routine clinical care. The ProPAED study will take a similar approach.

Similar to successfully completed adult studies, a pilot phase is foreseen to assure feasibility of the trial and to optimize study flow. Financial requirements during the main study phase will be kept to an absolute minimum, based on our experience and track-record in cost-control and consecutive patient recruitment.

Role of Funding Sources

Funds from the Dept. of Infectious Diseases at the University Children’s Hospital Basel are available to maintain the study centre. The heads of participating centres and the consultants of the respective emergency units will actively promote the successful completion of the study by making available the existing infrastructure and personnel of the respective emergency units. Brahms, the manufacturer of the PCT assay, will provide all assay related materials (Kryptor®, if not already available on-site) and kits and maintenance required for the measurements related to the study. Additional funds will be needed for ancillary projects. To exclude any conflicts of interest, no commercial sponsor will have any involvement in design and conduct of the studies (i.e., main project and ancillary projects), namely collection, management, analysis, and interpretation of the data; and preparation, decision to submit, review, or approval of the manuscript.

Publication & Authorship

Findings from this trial will be published in peer-reviewed journals. The “ProPAED” trial group as a whole will appear in an appendix in published manuscripts. Members of the steering committee, EAC, DSMB, co-investigators, statisticians, head paediatricians and laboratory heads of participating hospital will be listed in the appendix. Individuals who recruited >20 patients will be listed in the appendix as “Investigators”.

Criteria for authorship are as follows

All persons designated as authors should qualify for authorship. Each author should have participated sufficiently in the work to take public responsibility for the content. Authorship credit should be based on substantial contributions to 1) the intellectual content (i.e., conception and design, acquisition, analysis, and interpretation of data); and to 2) drafting of the article or revising it critically and prompt for important intellectual content; and 3) final approval of the version to be published. Two or three co-authorships slots for the main (primary) paper are assigned to each clinical centre after recruiting 50% or 100% of the agreed number of patients, respectively. Conditions 1, 2 and 3 must all be met for all authors. Potential conflicts of interest will be listed. Additional journal-specific regulations may apply.

The steering committee decides whether abstracts are to be submitted to conferences, and how the results are distributed if more than one manuscript is to be drafted. Providing that several manuscripts are to be drafted, a fair rotation among the participating centres of co-authorship slots will be done taking into consideration the number of patients enrolled and overall participation in the study. The clinical centre acknowledges that, due to the limited patient population in its treatment group, the data generated from its individual participation in the study and evaluation of its individual results, may not be sufficient to draw any meaningful scientific conclusions. In the event that the multi-centre publication has not been submitted within one year from the end of patient recruitment, notwithstanding the foregoing, each clinical centre may individually publish a manuscript regarding its individual results from the study, provided that the proposed publication is first reviewed by the steering committee.

## 2.4. TIMETABLE

| Start instruction of paediatricians | December 2008 |
| --- | --- |
| Start patient enrolment: feasibility phase | January 2009 |
| Start patient enrolment: study phase | February 2009 |
| Interim analysis | at 50% of patients included |
| End of patient recruitment | December 2009 |
| Follow-up (day 14), data entry completed | January 2010 |
| End of data analysis | April 2010 |
| Manuscript submitted, report to funding agencies | June 2010 |

## *2.5. SIGNIFICANCE AND OUTLOOK*

Adult studies have demonstrated the clinical and scientific impact of the proposed studies on the daily management of LRTI. These findings had a great impact in the medical community and received wide coverage by lay media. Because of the high prevalence and large need of health care resources for LRTI it has high relevance for the Swiss health care system and for public health in Switzerland, both for primary and hospital care.

Only a few new AB are in the development pipeline and AB overuse and resistance is thus a matter of serious concern. Microbiological diagnosis of CAP currently depends largely on 19th century methods. Conventional available blood tests (CRP, leukocyte count) also if used together with clinical signs and medical history as recommended by current guidelines are, based on data, largely insufficient to differentiate between LRTI from viral or bacterial origin. PCT-guidance improved the diagnosis (i.e., etiology) and, most importantly, proved to be useful in differentiating LRTI in need from those not in need of AB treatment.

For this trial, we anticipate improved diagnostic confidence, early detection of LRTI in need of antibiotic treatment, reduction of antibiotic misuse, shortening of the duration of antibiotic therapy and potentially reduction of the rate and duration of hospitalization..

The additional value of novel biomarkers in the careful clinical assessment shall be validated in this study in a large cohort of patients. With the help of these novel biomarkers we aim at delineation of a combination of signs and symptoms, reasonably predictive of LRTI in need of antibiotic treatment in settings with or without laboratory or radiological facilities. Thus, the envisioned prediction rules (e.g., scoring system) might be applicable in various clinical settings in industrialized and emerging countries.

Importantly, hospitalization is not only dependent on disease-related “medical” factors. Nursing care, social and hospital related aspects are at least as important in children. Their relevance for hospital admission rate and length will be explored this trial. Based on the large body of scientific evidence gathered by the ProPAED study, subsequent studies will be conceived. Thereby, we plan to combine the structured clinical assessment with diagnostic biomarkers including pivotal aspects of nursing care and social factors to conceive and aim for implementation of pre-emptive early discharge measures.

By synergizing all these important aspects, we expect a large potential to safely optimize health care resources. Thereby, this study will serve as a “proof-of-concept” for other diseases. A more tailored allocation of health care resources to patients at-risk and a better knowledge of non-medical extenuating circumstances is arguably *the* prerequisite for high quality health care in an era of cost-containment and emerging diagnosis related groups (“SwissDRG”).

##

## Prof. Dr. U.B. Schaad Dr. med. Jan Bonhoeffer

Medical Director Principle Investigator

University Children’s Hospital Basel University Children’s Hospital Basel

Date: Date:

## *2.6. REFERENCES*

## 1. Christ-Crain M, Muller B. Biomarkers in respiratory tract infections: diagnostic guides to antibiotic prescription, prognostic markers and mediators. Eur Respir J 2007;30(3):556-73.

## 2. Muller B, White JC, Nylen ES, Snider RH, Becker KL, Habener JF. Ubiquitous expression of the calcitonin-i gene in multiple tissues in response to sepsis. J Clin Endocrinol Metab 2001;86(1):396-404.

## 3. Domenech VS, Nylen ES, White JC, et al. Calcitonin gene-related peptide expression in sepsis: postulation of microbial infection-specific response elements within the calcitonin I gene promoter. J Investig Med 2001;49(6):514-21.

## 4. Christ-Crain M, Muller B. Procalcitonin and Pneumonia: Is it a Useful Marker? Curr Infect Dis Rep 2007;9(3):233-40.

## 5. Christ-Crain M, Stolz D, Bingisser R, et al. Procalcitonin guidance of antibiotic therapy in community-acquired pneumonia: a randomized trial. Am J Respir Crit Care Med 2006;174(1):84-93.

## 6. Muller B, Christ-Crain M, Nylen ES, Snider R, Becker KL. Limits to the use of the procalcitonin level as a diagnostic marker. Clin Infect Dis 2004;39(12):1867-8.

## 7. Simon L, Gauvin F, Amre DK, Saint-Louis P, Lacroix J. Serum procalcitonin and C-reactive protein levels as markers of bacterial infection: a systematic review and meta-analysis. Clin Infect Dis 2004;39(2):206-17.

## 8. Muller B. Endocrine aspects of critical illness. Ann Endocrinol (Paris) 2007;68(4):290-8.

## 9. Christ-Crain M, Jaccard-Stolz D, Bingisser R, et al. Effect of procalcitonin-guided treatment on antibiotic use and outcome in lower respiratory tract infections: cluster-randomised, single-blinded intervention trial. Lancet 2004;363(9409):600-7.

## 10. Don M, Valent F, Korppi M, et al. Efficacy of serum procalcitonin in evaluating severity of community-acquired pneumonia in childhood. Scand J Infect Dis 2007;39(2):129-37.

## 11. Masia M, Gutierrez F, Padilla S, et al. Clinical characterisation of pneumonia caused by atypical pathogens combining classic and novel predictors. Clin Microbiol Infect 2007;13(2):153-61.

## 12. Thayyil S, Shenoy M, Hamaluba M, Gupta A, Frater J, Verber IG. Is procalcitonin useful in early diagnosis of serious bacterial infections in children? Acta Paediatr 2005;94(2):155-8.

## 13. Madhi SA, Heera JR, Kuwanda L, Klugman KP. Use of procalcitonin and C-reactive protein to evaluate vaccine efficacy against pneumonia. PLoS Med 2005;2(2):e38.

## 14. Korppi M, Remes S. Serum procalcitonin in pneumococcal pneumonia in children. Eur Respir J 2001;17(4):623-7.

## 15. Korppi M, Remes S, Heiskanen-Kosma T. Serum procalcitonin concentrations in bacterial pneumonia in children: a negative result in primary healthcare settings. Pediatr Pulmonol 2003;35(1):56-61.

## 16. Michelow IC, Olsen K, Lozano J, et al. Epidemiology and clinical characteristics of community-acquired pneumonia in hospitalized children. Pediatrics 2004;113(4):701-7.

## 17. Moulin F, Raymond J, Lorrot M, et al. Procalcitonin in children admitted to hospital with community acquired pneumonia. Arch Dis Child 2001;84(4):332-6.

## 18. Toikka P, Irjala K, Juven T, et al. Serum procalcitonin, C-reactive protein and interleukin-6 for distinguishing bacterial and viral pneumonia in children. Pediatr Infect Dis J 2000;19(7):598-602.

## 19. McCormick A, Fleming D, Charlton J. Morbidity statistics from general practice, fourth national study 1991-1992. Office of Population Censuses and Surveys 1995;Series MB5 No 3.

## 20. Ashworth M, Charlton J, Latinovic R, Gulliford M. Age-related changes in consultations and antibiotic prescribing for acute respiratory infections, 1995-2000. Data from the UK General Practice Research Database. J Clin Pharm Ther 2006;31(5):461-7.

## 21. Baer G, Schaad UB, Heininger U. Clinical findings and unusual epidemiologic characteristics of human metapneumovirus infections in children in the region of Basel, Switzerland. Eur J Pediatr 2008;167(1):63-9.

## 22. Meury S, Zeller S, Heininger U. Comparison of clinical characteristics of influenza and respiratory syncytial virus infection in hospitalised children and adolescents. Eur J Pediatr 2004;163(7):359-63.

## 23. van Woensel JB, van Aalderen WM, Kimpen JL. Viral lower respiratory tract infection in infants and young children. Bmj 2003;327(7405):36-40.

## 24. Don M, Fasoli L, Paldanius M, et al. Aetiology of community-acquired pneumonia: serological results of a paediatric survey. Scand J Infect Dis 2005;37(11-12):806-12.

## 25. Tsolia MN, Psarras S, Bossios A, et al. Etiology of community-acquired pneumonia in hospitalized school-age children: evidence for high prevalence of viral infections. Clin Infect Dis 2004;39(5):681-6.

## 26. Cilla G, Onate E, Perez-Yarza EG, Montes M, Vicente D, Perez-Trallero E. Viruses in community-acquired pneumonia in children aged less than 3 years old: High rate of viral coinfection. J Med Virol 2008;80(10):1843-9.

## 27. Rossignoli A, Clavenna A, Bonati M. Antibiotic prescription and prevalence rate in the outpatient paediatric population: analysis of surveys published during 2000-2005. Eur J Clin Pharmacol 2007;63(12):1099-106.

## 28. Sharland M. The use of antibacterials in children: a report of the Specialist Advisory Committee on Antimicrobial Resistance (SACAR) Paediatric Subgroup. J Antimicrob Chemother 2007;60 Suppl 1:i15-26.

## 29. Briel M, Langewitz W, Tschudi P, Young J, Hugenschmidt C, Bucher HC. Communication training and antibiotic use in acute respiratory tract infections. A cluster randomised controlled trial in general practice. Swiss Med Wkly 2006;136(15-16):241-7.

## 30. Ehlken B, Ihorst G, Lippert B, et al. Economic impact of community-acquired and nosocomial lower respiratory tract infections in young children in Germany. Eur J Pediatr 2005;164(10):607-15.

## 31. Keren R, Chan E. A meta-analysis of randomized, controlled trials comparing short- and long-course antibiotic therapy for urinary tract infections in children. Pediatrics 2002;109(5):E70-0.

## 32. Kabra SK, Lodha R, Pandey RM. Antibiotics for community acquired pneumonia in children. Cochrane Database Syst Rev 2006;3:CD004874.

## 33. Haider BA, Saeed MA, Bhutta ZA. Short-course versus long-course antibiotic therapy for non-severe community-acquired pneumonia in children aged 2 months to 59 months. Cochrane Database Syst Rev 2008(2):CD005976.

## 34. Piaggio G, Elbourne DR, Altman DG, Pocock SJ, Evans SJ. Reporting of noninferiority and equivalence randomized trials: an extension of the CONSORT statement. Jama 2006;295(10):1152-60.

## 35. Glouberman S, Mintzberg H. Managing the care of health and the cure of disease--Part I: Differentiation. Health Care Manage Rev 2001;26(1):56-69; discussion 87-9.

## 36. Glouberman S, Mintzberg H. Managing the care of health and the cure of disease--Part II: Integration. Health Care Manage Rev 2001;26(1):70-84; discussion 7-9.

## 37. Little P, Rumsby K, Kelly J, et al. Information leaflet and antibiotic prescribing strategies for acute lower respiratory tract infection: a randomized controlled trial. Jama 2005;293(24):3029-35.

## 38. Watson L, Little P, Moore M, Warner G, Williamson I. Validation study of a diary for use in acute lower respiratory tract infection. Fam Pract 2001;18(5):553-4.

## 39. Le Saux N, Bjornson C, Pitters C. Antimicrobial use in febrile children diagnosed with respiratory tract illness in an emergency department. Pediatr Infect Dis J 1999;18(12):1078-80.

## 40. Stone S, Gonzales R, Maselli J, Lowenstein SR. Antibiotic prescribing for patients with colds, upper respiratory tract infections, and bronchitis: A national study of hospital-based emergency departments. Ann Emerg Med 2000;36(4):320-7.

## 41. Huang N, Morlock L, Lee CH, Chen LS, Chou YJ. Antibiotic prescribing for children with nasopharyngitis (common colds), upper respiratory infections, and bronchitis who have health-professional parents. Pediatrics 2005;116(4):826-32.

## Appendix 1: Patient information and consent form

**Patienteninformation**

Liebe Eltern,

wir möchten Sie einladen an einer Studie teilzunehmen, von der wir glauben, dass sie uns hilft Ihr Kind in Zukunft noch besser behandeln zu können.

**Allgemeine Informationen zur klinischen Studie**

Wir führen zurzeit eine Studie an über 300 Patienten durch, die zum Ziel hat, die Betreuung von Patienten mit akuten Infektionen der unteren Atemwege (wie bei Ihrem Kind) zu verbessern. Ein unterer Atemwegsinfekt kann durch Viren oder Bakterien verursacht werden. Nur bakterielle Infekte, nicht aber virale, sollten mit Antibiotika behandelt werden. Für Ärzte ist es schwierig herauszufinden, ob tatsächlich Bakterien am Infekt beteiligt sind, und ob und wie lange deshalb ein Antibiotikum verschrieben werden sollte. Seit kurzem gibt es ein neues Nachweisverfahren für Infektionen – den Nachweis von Procalcitonin im Blut. Es hilft bei der Entscheidung, ob Ihr Kind von einem Antibiotikum profitieren kann und wie lange dieses eingenommen werden sollte.

**Ziel der Studie**

In dieser Studie untersuchen wir an mehreren Spitälern, ob Atemwegsinfektionen, die mit einem Antibiotikum behandelt werden müssen, dank dem neuen Nachweisverfahren früher erkannt und damit frühzeitiger behandelt werden können. Wir wollen auch untersuchen, ob mit dem neuen Bluttest die Anzahl und Dauer von Antibiotikatherapien vermindert werden kann. Die Verminderung der Verschreibung von Antibiotika ist deshalb erstrebenswert, weil die übermässige und zu lange Einnahme nicht nur eine Belastung der Familie bedeutet, sondern auch die die Resistenzentwicklung der Bakterien und das Auftreten von Nebenwirkungen begünstigt.

**Auswahl der Studienteilnehmer**

Sie wurden bezüglich Teilnahme an dieser Studie angefragt, weil Ihr Kind einen Infekt der unteren Luftwege (Lungenentzündung, Bronchitis oder Bronchiolitis) hat und der behandelnde Arzt nun entscheiden muss, ob er Ihrem Kind ein Antibiotikum verschreiben soll oder nicht.

**Datenschutz und freiwillige Teilnahme**

Alle im Rahmen dieser Studie erhobenen Patientendaten werden entsprechend der medizinischen Schweigepflicht absolut vertraulich und anonymisiert behandelt. Die Studienteilnahme ist freiwillig. Sie können Ihr Einverständnis zur Studienteilnahme Ihres Kindes jederzeit zurückziehen, ohne Angabe von Gründen und ohne, dass dadurch Nachteile für die weitere medizinische Betreuung entstehen. In diesem Falle würde Ihr Kind zur Sicherheit abschliessend medizinisch untersucht werden. Sie können jederzeit uneingeschränkte Einsicht in alle von uns erhobenen Studiendaten und Befunde erhalten.

**Studienablauf**

Ihr Kind wird nach dem Zufallsprinzip zu einer Behandlung des Infektes mit oder ohne Hilfe dieses Bluttests eingeteilt. Für Sie bedeutet dies folgendes:

Möglichkeit 1: Ihr Kind wird der Gruppe mit üblicher Behandlung zugeteilt. Der behandelnde Arzt entscheidet nach einer Blutentnahme und den von ihm notwendig erachteten Massnahmen, ob er ein Antibiotikum verschreiben möchte und wenn ja, für wie lange. Behandlung und Nachkontrollen erfolgen wie gewöhnlich, wobei an den Tagen 3 und 5 eine Blutkontrolle durchgeführt wird. Falls Ihr Kind nicht hospitalisiert werden muss, und es Ihrem Kind im Verlauf schlechter geht, bitten wir Sie unverzüglich mit unserer Notfallstation Kontakt aufzumehmen, damit wir Ihr Kind nachkontrollieren und bestmöglich betreuen können.

Möglichkeit 2: Ihr Kind wird der Gruppe mit Bestimmung des Procalcitonins zugeteilt. Der Arzt wird das Ergebnis des Procalcitonin-Tests in der Entscheidung, ein Antibiotikum zu verordnen, berücksichtigen. Ihr Kind erhält ein Antibiotikum, wenn der Laborwert Procalcitonin erhöht ist. Bei tiefem Procalcitonin muss zur Sicherheit innerhalb von 6 – 24 Stunden eine zweite Blutentnahme durchgeführt werden, um einen verspäteten Anstieg zu erkennen. Ist das Procalcitonin erhöht oder geht es Ihrem Kind schlechter, wird ein Antibiotikum verschrieben. Die Behandlung erfolgt wie gewöhnlich, wobei an den Tagen 3 und 5 eine Blutkontrolle durchgeführt wird. Falls Ihr Kind ein Antibiotikum bekommen hat und der Procalcitonin-Wert stark abgesunken ist, kann die Antibiotikatherapie bereits nach wenigen Tagen gestoppt werden. Falls Ihr Kind nicht hospitalisiert werden muss, und es Ihrem Kind im Verlauf schlechter geht, bitten wir Sie unverzüglich mit unserer Notfallstation Kontakt aufzumehmen, damit wir Ihr Kind nachkontrollieren und bestmöglich betreuen können.

Unabhängig von der Gruppe, der Ihr Kind zugeteilt ist, bitten wir Sie ein einfaches, vorgefertigtes Tagebuch zu führen, in dem Sie jeden Tagen Fragen zum Gesundheitszustandes Ihres Kindes ankreuzen. Wir werden auch nach 14 Tagen ein 5-minütiges Telefoninterview durchführen, in dem wir überprüfen, ob beide Gruppen gleich gut behandelt wurden, und der Infekt in beiden Gruppen gleich gut abgeheilt ist. Sie werden über Rückfälle und ob Sie mit Ihrem Kind nochmals den behandelnden Arzt oder das Spital aufsuchen mussten, befragt.

Der behandelnde Spitalarzt hat während der gesamten Studie freie Wahl bezüglich sämtlicher diagnostischer Massnahmen, weiterer Behandlungen sowie der Wahl des Antibiotikums.

**Alternative Behandlungsmethoden**

Wenn Sie nicht an der Studie teilnehmen wollen, erhalten Sie die gleiche medizinische Betreuung, ohne irgendwelche Nachteile.

**Vorteile durch die Studie**

Mit der Studienteilnaehme ermöglichen Sie es, wertvolle Informationen zum Nutzen des Procalcitonins bei Infektionen zu gewinnen. Ihr Kind und zukünftige weitere Kinder können damit von einer gezielteren Antibiotikaverordnung profitieren. Mittels Procalcitonin-Messung lässt sich eine Atemwegsinfektion, die mit einem Antibiotikum behandelt werden muss frühzeitig erkennen und behandeln. Andererseits lässt sich eine unnötige antibiotische Therapie wahrscheinlich vermeiden. Die optimale Dauer der Antibiotikatherapie lässt sich wahrscheinlich auch verkürzen. Zusätzlich hoffen wir zu zeigen, dass Kinder und deren Eltern mit Hilfe des Procalcitonintests weniger häufig und weniger lange im Spital sein müssen. Eine gezieltere antibiotische Therapie kann bei Ihrem Kind und anderen Kindern die Resistenzentwicklung gegen Antibiotika vermindern und das Risiko von antibiotikabedingten Nebenwirkungen wie Durchfall, Bauchschmerzen oder Erbrechen reduzieren als auch zur Kostenreduktion im Gesundheitswesen beitragen.

**Nachteile durch die Studie**

Während der Studie werden zusätzliche Blutentnahmen durchgeführt. Dabei werden Ihrem Kind jeweils 5 ml Blut entnommen. Das ist eine kleine Menge, die sogar bei den meisten Neugeborenen unbedenklich ist. Es besteht die Möglichkeit, dass aufgrund eines niedrigen Procalcitonin- Wertes kein Antibiotikum verschrieben wird, obwohl die Verordnung eines Antibiotikums sich nachträglich doch als notwendig erweisen kann. Eine verzögerte Antibiotikatherapie sowie der vorzeitige Abbruch der Antibiotikatherapie bei Infekten kann das Risiko von Komplikationen wie einem schweren Infekt oder einer Blutvergiftung erhöhen. Dies ist aber aufgrund unserer Erfahrung an über 1000 Erwachsenen mit dem Bluttest Procalcitonin am Universitätsspital Basel sehr unwahrscheinlich. Um eine gute Überwachung zu gewähren, werden zu Beginn der Studie engmaschige Kontrollen und Blutentnahmen durchgeführt.

**Vergütung von Auslagen der Studienteilnehmer**

Alle erwähnten Untersuchungen dieser Studie sind kostenlos für Sie. Weder Ihnen noch der Krankenkasse entstehen im Zusammenhang mit dieser Studie zusätzliche Kosten.

**Versicherung**

Sollte Ihr Kind im Rahmen dieser Studie entgegen unseren Erwartungen in Folge der zugeordenetn Abklärung und Behandlung einen Schaden erleiden, ist das Universitäts- Kinderspital beider Basel haftbar. Zu diesem Zweck haben die Studienverantwortlichen eine Versicherung bei der RIMAS abgeschlossen. Stellen Sie während oder nach dem klinischen Versuch gesundheitliche Probleme oder andere Schäden fest, so wenden Sie sich bitte an die Studienleitung. Diese weiss Bescheid über die geltende Gesetzgebung, verfügt über die entsprechenden Unterlagen und wird für Sie die notwendigen Schritte einleiten.

**Studienleitung**

Universitäts-Kinderspital beider Basel, Prof. Dr. U. Heininger/ Dr. J. Bonhoeffer.

Kontaktadressen Basel/Aarau siehe Patiententagebuch.

| **Initialen des Untersuchers**  (Vor-/Nachnahme) | **Patientenkleber** | **Studien-Nr.** |
| --- | --- | --- |

**Schriftliche Einverständniserklärung des Patienten zur Teilnahme an der ProPAED-Studie**

- Ich wurde vom unterzeichnenden Arzt mündlich und schriftlich über die Ziele, den Ablauf der Studie mit dem Blutmarker Procalcitonin, über die zu erwartenden Wirkungen, über mögliche Vor- und Nachteile sowie über eventuelle Risiken informiert.
- Ich habe die zur oben genannten Studie abgegebene schriftliche Patienteninformation gelesen und verstanden. Meine Fragen im Zusammenhang mit der Teilnahme an dieser Studie sind mir zufrieden stellend beantwortet worden. Ich kann die schriftliche Patienteninformation behalten und erhalte eine Kopie meiner schriftlichen Einverständniserklärung.
- Ich hatte genügend Zeit, um meine Entscheidung zu treffen.
- Ich bin darüber informiert, dass eine Versicherung Schäden deckt, falls solche im Rahmen der Studie auftreten sollten.
- Ich bin einverstanden, dass die zuständigen Fachleute der Studienleitung, der Behörden und der Ethikkommission zu Prüf- und Kontrollzwecken in die Originaldaten meines Kindes Einsicht nehmen dürfen, jedoch unter strikter Einhaltung der Vertraulichkeit.
- Im Interesse der Gesundheit meines Kindes kann der Prüfarzt jederzeit einen Studienausschluss veranlassen.
- Ich orientiere den Prüfarzt über die gleichzeitige Behandlung bei einem anderen Arzt sowie über die Einnahme von Medikamenten (vom Arzt verordnete und selbst gekaufte).
- Ich bin bereit während 14 Tagen ein einfaches, vorgefertigtes Tagebuch zu führen, um damit den Heilungsverlauf zu dokumentieren.
- Falls mein Kind ambulant (teilweise) behandelt werden kann, bin ich bereit zu 2 Kontrolluntersuchungen am Tag 3 und 5 zu kommen, um eine gute Überwachung zur Sicherheit meines Kindes sicher zu stellen.
- Falls mein Kind (teilweise) ambulant behandelt werden kann, werde ich im Falle einer Verschlechterung des Zustandes meines Kindes sofortigen Kontakt mit der Studienleitung aufnehmen und ggf. zu einer Kontrolluntersuchung kommen.
- Ich bin einverstanden damit, nach 2 Wochen für ein kurzes Telefoninterview angerufen zu werden.
- Ich bin damit einverstanden, dass alle Blutproben, die bei meinem Kind während der Studie abgenommen wurden bis Abschluss der Gesamt-Studie eingefroren werden und anstatt das Restblut zu verwerfen, rückwirkend ggf. weitere, Blutmarker bestimmt werden, die eine zusätzliche Verbesserung der Diagnose von Atemweginfekten in der Zukunft ermöglichen.

Ort, Datum Unterschrift der Eltern/ Fürsorgeperson

**Bestätigung des Prüfarztes**

Hiermit bestätige ich, dass ich den Eltern Wesen, Bedeutung und Tragweite der Studie erläutert habe. Ich versichere, alle im Zusammenhang mit dieser Studie stehenden Verpflichtungen zu erfüllen. Sollte ich zu irgendeinem Zeitpunkt während der Durchführung der Studie von Aspekten erfahren, welche die Bereitschaft zur Teilnahme an der Studie beeinflussen könnten, werde ich die Eltern umgehend darüber informieren.

Ort, Datum Unterschrift des Prüfarztes

Studienleitung: Universitäts-Kinderspital beider Basel UKBB, Prof. Dr. U. Heininger/ Dr. J. Bonhoeffer

Kontaktadressen Basel/Aarau siehe Patiententagebuch.

**Bitte 2 x kopieren (1 x Patient, 1 x KG), Original an Datenzentrum per Post schicken!**

## Appendix 2: Patient Diary

| **Initialen des Untersuchers**  (Vor-/Nachnahme) | **Patienten-Nr.** | **Studien-Nr.** |
| --- | --- | --- |

**Patienten-Tagebuch**

**Bitte beantworten Sie folgende Fragen am Abend jedes Tages und bewerten Sie jede mit einer der folgenden Zahlen:**

| **0= Normal/nicht zutreffend** | **4= Schweres Problem** |
| --- | --- |
| **1= Praktisch kein Problem** | **5= Sehr schweres Problem** |
| **2= Kleines Problem** | **6= So schwer, wie es sein kann** |
| **3= Mittel schweres Problem** |  |

| **Tag** | **1** | **2** | **3** | **4** | **5** | **6** | **7** | **8** | **9** | **10** | **11** | **12** | **13** | **14** |
| --- | --- | --- | --- | --- | --- | --- | --- | --- | --- | --- | --- | --- | --- | --- |
| **Husten?** |  |  |  |  |  |  |  |  |  |  |  |  |  |  |
| **Schleim beim Husten?** |  |  |  |  |  |  |  |  |  |  |  |  |  |  |
| **Mühe zu atmen?** |  |  |  |  |  |  |  |  |  |  |  |  |  |  |
| **Schlaf gestört ?** |  |  |  |  |  |  |  |  |  |  |  |  |  |  |
| **Normale Aktivität?** |  |  |  |  |  |  |  |  |  |  |  |  |  |  |
| **Krankheitsgefühl?** |  |  |  |  |  |  |  |  |  |  |  |  |  |  |

**Bitte schreiben Sie auf, wie oft Ihr Kind ein Medikament (Saft, Tablette, Zäpfchen) gegen Fieber erhalten hat:**

| **Tag** | **1** | **2** | **3** | **4** | **5** | **6** | **7** | **8** | **9** | **10** | **11** | **12** | **13** | **14** |
| --- | --- | --- | --- | --- | --- | --- | --- | --- | --- | --- | --- | --- | --- | --- |
| **Anzahl der Dosen** |  |  |  |  |  |  |  |  |  |  |  |  |  |  |

**Bitte tragen Sie die Höhe des Fiebers am Abend ein:**

| **Tag** | **1** | **2** | **3** | **4** | **5** | **6** | **7** | **8** | **9** | **10** | **11** | **12** | **13** | **14** |
| --- | --- | --- | --- | --- | --- | --- | --- | --- | --- | --- | --- | --- | --- | --- |
| **Temperatur (°C)** |  |  |  |  |  |  |  |  |  |  |  |  |  |  |

**Bitte kreuzen die entsprechenden Felder an, wenn die Aussage zutrifft: x für ja**

| **Tag** | **1** | **2** | **3** | **4** | **5** | **6** | **7** | **8** | **9** | **10** | **11** | **12** | **13** | **14** |
| --- | --- | --- | --- | --- | --- | --- | --- | --- | --- | --- | --- | --- | --- | --- |
| **Stationär im Spital?** |  |  |  |  |  |  |  |  |  |  |  |  |  |  |
| **Sauerstofftherapie?** |  |  |  |  |  |  |  |  |  |  |  |  |  |  |
| **Intensivstation?** |  |  |  |  |  |  |  |  |  |  |  |  |  |  |
| **Künstlich beatmet?** |  |  |  |  |  |  |  |  |  |  |  |  |  |  |
| **Kann Ihr Kind trinken?** |  |  |  |  |  |  |  |  |  |  |  |  |  |  |
| **Antibiotikum**  **- verschrieben?** |  |  |  |  |  |  |  |  |  |  |  |  |  |  |
| **- genommen?** |  |  |  |  |  |  |  |  |  |  |  |  |  |  |
| **Erbrechen?** |  |  |  |  |  |  |  |  |  |  |  |  |  |  |
| **Durchfall?** |  |  |  |  |  |  |  |  |  |  |  |  |  |  |
| **Ausschlag?** |  |  |  |  |  |  |  |  |  |  |  |  |  |  |
| **Schmerzen?** |  |  |  |  |  |  |  |  |  |  |  |  |  |  |
| **Arbeitsausfall eines Elternteils?** |  |  |  |  |  |  |  |  |  |  |  |  |  |  |
| **Ausfall Kindergarten/ Schule/Lehre** |  |  |  |  |  |  |  |  |  |  |  |  |  |  |

| **Initialen des Untersuchers**  (Vor-/Nachnahme) | **Patienten-Nr.** | **Studien-Nr.** |
| --- | --- | --- |

**Wie stark ist Ihr Kind in seinem Alltag durch den Atemwegsinfekt eingeschränkt (von 0 - 100%)?**

100

So sehr

wie nie zuvor

Gar nicht

**Tag 1**

0

10

20

30

40

50

60

70

80

900

100

So sehr

wie nie zuvor

Gar nicht

**Tag 2**

0

10

20

30

40

50

60

70

80

900

100

So sehr

wie nie zuvor

Gar nicht

**Tag 3**

0

10

20

30

40

50

60

70

80

900

100

So sehr

wie nie zuvor

Gar nicht

**Tag 4**

0

10

20

30

40

50

60

70

80

900

100

So sehr

wie nie zuvor

Gar nicht

**Tag 5**

0

10

20

30

40

50

60

70

80

900

100

So sehr

wie nie zuvor

Gar nicht

**Tag 6**

0

10

20

30

40

50

60

70

80

900

100

So sehr

wie nie zuvor

Gar nicht

**Tag 7**

0

10

20

30

40

50

60

70

80

900

100

So sehr

wie nie zuvor

Gar nicht

**Tag 8**

0

10

20

30

40

50

60

70

80

900

100

So sehr

wie nie zuvor

Gar nicht

**Tag 9**

0

10

20

30

40

50

60

70

80

900

100

So sehr

wie nie zuvor

Gar nicht

**Tag 10**

0

10

20

30

40

50

60

70

80

900

100

So sehr

wie nie zuvor

Gar nicht

**Tag 11**

0

10

20

30

40

50

60

70

80

900

100

So sehr

wie nie zuvor

Gar nicht

**Tag 12**

0

10

20

30

40

50

60

70

80

900

100

So sehr

wie nie zuvor

Gar nicht

**Tag 13**

0

10

20

30

40

50

60

70

80

900

100

So sehr

wie nie zuvor

Gar nicht

**Tag 14**

0

10

20

30

40

50

60

70

80

900

**Bitte das Tagebuch zukleben und an das Kinderspital zurücksenden (gratis) !!**

Universitäts- Kinderspital Kantonsspital Aarau:

Beider Basel:

**Bei Fragen zu Terminen: Bei Fragen zu Terminen:**

Telefon: 061 685 65 47 Telefon: 062 838 49 19

**Bei medizinischen Fragen: Bei medizinischen Fragen:**

Telefon: 061 685 67 88 Telefon: 062 838 57 34

Bitte keinen

Absender angeben !!!

Porto bezahlt Ihr

Kinderspital für Sie

**☺☺☺**

**UKBB**

**Universitäts- Kinderspital**

**Beider Basel**

**Infektiologie**

**Postfach**

**4005 Basel**

## Appendix 3: CRF 1- Baseline inclusion

| **Initialen des Untersuchers**  (Vor-/Nachnahme) | **Patienten-Nr.** | **Studien-Nr.** |
| --- | --- | --- |

**Einschluss, Datum** **.****.**(dd.mm.jj) **,** **Zeit** **.**(hh.mm)

| **Einschlusskriterien*** | **Nein** | **Ja** | **Anzahl Tage** |  | **Ausschlusskriterien** |  |  |
| --- | --- | --- | --- | --- | --- | --- | --- |
| Fieber |  |  |  |  |  | **Nein** | **Ja** |
| **A ( ≥ 1 Symptom)** |  |  |  |  | Kein Einverständnis | -- |  |
| Husten |  |  |  |  | Sprachverständnis unzureichend |  |  |
| Sputum |  |  |  |  | Anderer Grund: | -- | -- |
| Inspiratorischer Schmerz |  |  |  |  |  |  |  |
| Trinkschwäche |  |  |  |  | Hosp. mit Pneumonie vor < 2 Wo |  |  |
| **B ( ≥ 1 Zeichen)** | -- | -- |  |  | Pseudokrupp |  |  |
| Tachypnoe |  |  |  |  | Cystische Fibrose |  |  |
| Dyspnoe^#^ |  |  |  |  | Schwere Immunsuppression |  |  |
| Bilaterales Giemen |  |  |  |  | - Bek. Neutropenie (< 1000x10e9) |  |  |
| Rasselgeräusche | -- | -- |  |  | - Bekannte CD4 < 350x10e9) |  |  |
| - endinspiratorisch |  |  |  |  | - Immunsuppressive Therapie |  |  |
| - Knisterrrasseln |  |  |  |  | Chronische Infektion |  |  |
| Abgeschw. Atemgeräusch |  |  |  |  |  |  |  |
| Bronchialatmen |  |  |  |  |  |  |  |
| Pleurareiben |  |  |  |  |  |  |  |

* Die **Kombination Fieber, Trinkschwäche, Tachypnoe** ist unspezifisch für einen Infekt der unteren Luftwege. daher muss in diesem Fall entweder

die Tachypnoe nach effektiver Antipyrese persistieren, oder ein weiteres Symptom oder Zeichen vorhanden sein.

^#^ stossende Atmung, Einziehungen, verlängertes Exspirium, Nasenfügeln.

**Wie lautet Ihre initiale Diagnose?** ( mehr als eine Antwort (z.B. Bronchitis und Pneumonie) zulässig)

| **Bronchitis** |  | **Bronchiolitis** |  | **Pneumonie** |  |
| --- | --- | --- | --- | --- | --- |

| **Basisdaten** | | |
| --- | --- | --- |
| Geb.-Dat. .. | Geschlecht  w m | .  kg |
| Temp. .°C | Puls  / min |  |
| AF | SpO2  % mit  L Sauerstoff | |
| NPS abgenommen | Nein Ja Keim_______________ | |
| BK abgenommen | Nein Ja Keim_______________ | |

| **Relevante vorbestehende Behandlung** | | | |
| --- | --- | --- | --- |
| **Anbehandelt mit Antibiotika** | Nein  Ja, seit .., Name _________________Dosis:  mg/d | | |
| **Allergien gegen Antibiotika** | Nein Ja:_____________________________________ | | |
| **Wieviele IdULW wurden im letzten Jahr mit AB behandelt?** |  | | |
| **Impfstatus** (ggf. Impfpass heute erbitten und bei Kontrolle ergänzen) | Pneumokokken  0x 1x 2x 3x 4x | H.influenzae  0x 1x 2x 3x 4x | Unbekannt |

| **Soziale Situation** | |
| --- | --- |
| **Betreuung** | Zuhause Tagesstätte/ Heim Kindergarten Schule / Lehre |
| **Ausbildung der Mutter** | Schule  Lehre  Fachhochschule  Universität |
| **Geschwister** | Anzahl Wievieltes Kind |

**Bitte sofort ans Datenzentrum faxen, vielen Dank: 061 685 60 12**

| **Initialen des Untersuchers**  (Vor-/Nachnahme) | **Patientenkleber** | **Studien-Nr.** |
| --- | --- | --- |

**Telefon der Eltern: Kinderarzt:**

**Name:**

Zu Hause: (+ ) -  Tel.: (+ ) -

Mobiltelefon: (+ ) -  Fax: (+ ) -

**Kontrollen (ambulant oder stationär) geplant am:**

**Datum** **.****.**(dd.mm.jj) **,** **Zeit** **.**(hh.mm) (6-24 Stunden wenn PCT < 0.25 ng/ml)

**Datum** **.****.**(dd.mm.jj) **,** **Zeit** **.**(hh.mm) (Tag 3, CRF ausfüllen, PCT messen)

**Datum** **.****.**(dd.mm.jj) **,** **Zeit** **.**(hh.mm) (Tag 5 CRF ausfüllen, PCT messen)

**Telefoninterview durch Datenzentrum:**

**Datum** **.****.**(dd.mm.jj) **,** **Zeit** **.**(hh.mm) (Tag 14; bzw. wenn Sa/ So: Tag 15/ 16)

**Ort der Kontrolle:**

**Basel: Universitäts-Kinderspital beider Basel (UKBB), Notfallstation Basel Stadt**

**Römergasse 8, 4048 Basel, Tel: 061 685 65 47**

**Aarau: Kinderklinik Notfallstation, Haus 9, Kantonspital,**

**Tellstrasse 1, 5001 Aarau, Tel: 062 838 49 19**

**Bitte sofort an Datenzentrum faxen und dem Patienten eine Kopie**

**mitgeben, Original bleibt in KG, vielen Dank: 061 685 60 12**

**Procedere Tag 1**

| **Initialen des Untersuchers**  (Vor-/Nachnahme) | **Patienten-Nr.** | **Studien-Nr.** |
| --- | --- | --- |

**□ Info Studien-Team**

**□ Einverständniserklärung**

**□ Labor □ NPS □ Röntgen Thorax**

**Hospitalisiert Ja □ Nein □**

□ Allgemeinzustand

□ Sauerstoffbedarf

□ Flüssigkeit via MS/ IV

□ **Procalcitonin-Gruppe**

- **Guideline Gruppe**

**Antibiotische Therapie gemäss ProCT (ng/ml):**

**Keine antibiotische Therapie bei V.a.**

□ Bronchitis

□ Bronchiolitis

**Indikation für antibiotische Therapie:**

□ V.a. bakt. Pneumonie

□ Bronchitis + V.a. bakt. Pneumonie

□ Bronchiolitis + V.a. bakt. Pneumonie

□ < 0.1 KEIN AB!

□ 0.1 - 0.25 kein AB

□ > 0.25 AB start/weiter

□ > 0.5 AB START/WEITER

**ProCT-Kontrolle**

**nach 12-24h**

**Kriterien für antibiotische Therapie**

□ Anamnese (inf. d. OLW 🡪 Fieber + AZ ↓ + Tachypnoe)

□ Klinik (Auskultation, schlechter AZ, septisch)

□ Bildgebung (Infiltrat und/oder Erguss)

**Overruling?**

- Kardio-Respir. Instabilität
- Schwere Sepsis/ Schock
- Rücksprache mit Basel bzw. Aarau

BS: 076 33 99 691 AR: 076 33 99 694

**Ambulant/ Entlassen**

**Antibiotische Behandlung**

□ Amoxicillin/Clavulansäure

80-90 mg/kg/d, in 2 ED, PO

□ Clarithromycin

15mg/kg/d in 2 ED, PO

**Hospitalisiert**

**Antibiotische Behandlung**

**PO**

□ Amoxicillin/Clavulansäure

80-90 mg/kg/d, in 2 ED, PO

□ Clarithromycin

15mg/kg/d, in 2 ED, PO

**IV**

□ Amoxicillin/Clavulansäure

90(-120) mg/kg/d, in 3(-4) ED, IV

□ Clarithromycin

15mg/kg/d, in 2 ED, IV

**Kriterien für IV**

□ Allgemeinzustand

□ Orale Aufnahme ↓

□ Enterale Resorption ↓

□ Ansprechen auf AB PO↓

**Ambulant**

□Amoxicillin/Clavulansäure

80-90mg/kg/d, in2 ED,PO

□Clarithromycin

15mg/kg/d, in 2 ED PO

**Hospitalisiert**

**Antibiotische Behandlung**

**PO**

□ Amoxicillin/Clavulansäure

80-90 mg/kg/d, in 2 ED, PO

□ Clarithromycin

15mg/kg/d, in 2 ED, PO

**IV**

□ Amoxicillin/Clavulansäure

90 (-120) mg/kg/d, in 3(-4)ED, IV

□ Clarithromycin

15mg/kg/d, in 2 ED, IV

**Kriterien für IV**

□ Allgemeinzustand

□ Orale Aufnahme ↓

□ Enterale Resorption ↓

□ Ansprechen auf AB PO↓

**Dauer der Therapie**

□ 10 Tage □ ≥ 14 Tage (z.B. Erguss)

**Dauer der Therapie**

**je nach PCT am Tag 3 und 5**

**Bitte sofort ans Datenzentrum faxen, vielen Dank: 061 685 60 12**

## Appendix 4: CRF 2 – Day 3

**Tag 3, Datum** **.****.**(dd.mm.jj)

| **Untersucher Initialen**  (Vor-/Nachnahme) | **Patienten-Nr.** | **Studien-Nr.** |
| --- | --- | --- |

**Aktuelle Diagnose?** ( mehr als eine Antwort (z.B. Bronchitis und Pneumonie) zulässig)

| **Bronchitis** |  | **Bronchiolitis** |  | **Pneumonie** |  |
| --- | --- | --- | --- | --- | --- |

| **Temperatur** **.**°C | **AF** /min | **SaO2** % mit LO_2_/min |
| --- | --- | --- |
| **Puls** / min |  |  |
|  |  |  |
| **Ambulant?** | Ja, seit .. |  |
| **Hospitalisiert?** | Ja, seit .. |  |
|  |  |  |
| **Antibiotische Therapie *per os*** | Ja, von Anfang an  Ja, seit .., mit __________________  Ja, am .. Wechsel von i.v. auf p.o. Therapie  Nein, immer noch i.v  Nein, keine antibiotische Therapie seit .. | |

| **Sekundäre Endpunkte** | | | |
| --- | --- | --- | --- |
| **Erneute Dyspnoe**  **(nach Besserung)** | Nein  Ja, seit .. |  |  |
| **Tachypnoe nach Tag 1** | Nein  Ja, seit ..  Kein Effekt von Salbutamol | **SpO2 <90%**  **nach Tag 1** | Nein  Ja, seit ..  Kein Effekt von Salbutamol |
| **Fieber >38°C nach Tag 1** | Nein  Ja, bis .. | **Tod** | Nein  Ja, am .. |
| **Komplikationen der Pneumonie** | Nein  Ja, seit .. | ARDS  Pleuraerguss (Punktion)  Sepsis / Schock  Pleuraempyem  Lungenabszess  Andere_____________ | |
| **Andere Komplikationen** | Nein  Ja, seit .. | Otitis media  Meningo-Encephalitis  Andere:__________________________________ | |
| **Antibiotische Therapie wegen „Andere Komplikation“?** | Nein  Ja | von .. bis ..  mit __________________ | |
| **Intensivstation?** | Nein  Ja  von ..  bis .. | Beatmet für  Tage  Grund für IPS: | |
| **Gründe für Hospitalisation länger als IV AB Therapie (Total 100%)** | Medizinische ____%  (z.B. O_2_ Bedarf, Flüssigkeit)  Pflegerische __%  (z.B. professionelle Pflege nötig) | Elterliche %  (z.B. Wunsch/Sorge der Eltern)  Soziale %  (z.B. Austritt nach Hause/Heim nicht möglich) | |

| (Vor-/Nachnahme) | **Patienten-Nr.** | **Studien-Nr.** |
| --- | --- | --- |

**Procedere Tag 3**

**Impfstatus, falls nicht auf Tag 1 CRF**

□ 1 x Pneumokokken □ 2 x Pneumokokken □ 3 x Pneumokokken □ 4 x Pneumokokken

□ 1 x HiB □ 2 x HiB □ 3 x HiB □ 4 x HiB

- **Blutentnahme ( s. Laborblatt Tag 3)**

**Overruling?**

Kardio-Respir. Instabilität

Schwere Sepsis/ Schock

Rücksprache mit Basel bzw. Aarau

BS: 076 33 99 691 AR :076 33 99 694

□ **Guideline-Gruppe**

□ **Procalcitonin-Gruppe**

| **Bitte incl. Blatt „Tag 3“ sofort ans Datenzentrum faxen, vielen Dank: 061 685 60 12** |
| --- |

**Gabe der Antibiotika gemäss Guidelines:**

**□ Keine antibiotische Therapie**

**□ Therapie der CAP: siehe Procedere Tag 1**

**□ Andere Gründe für AB:_________________**

□ < 0.1 KEIN AB!

□ 0.1 - 0.25 kein AB

□> 0.5 AB START/WEITER

**Tagebuch prüfen und kopieren (AE?)**

**Therapie: siehe Procedere Tag 1**

**Kontrolle Tag 5**

**Antibiotische Therapie gemäss ProCT(ng/ml)**

□ > 0.25 AB Start/ weiter

## Appendix 5 CRF 3 – Day 5

**Tag 5, Datum** **.****.**(dd.mm.jj)

| **Untersucher Initialen**  (Vor-/Nachnahme) | **Patienten-Nr.** | **Studien-Nr.** |
| --- | --- | --- |

**Aktuelle Diagnose? ( mehr als eine Antwort (z.B. Bronchitis und Pneumonie) zulässig)**

| **Bronchitis** |  | **Bronchiolitis** |  | **Pneumonie** |  |
| --- | --- | --- | --- | --- | --- |

| **Temperatur** **.**°C | **AF** /min | **SaO2** % mit LO_2_/min |
| --- | --- | --- |
| **Puls** / min |  |  |
|  |  |  |
| **Ambulant?** | Ja, seit .. |  |
| **Hospitalisiert?** | Ja, seit .. |  |
|  |  |  |
| **Antibiotische Therapie *per os*** | Ja, von Anfang an  Ja, seit .., mit __________________  Ja, am .. Wechsel von i.v. auf p.o.Therapie  Nein, immer noch i.v  Nein, keine antibiotische Therapie seit .. | |

| **Sekundäre Endpunkte** | | | |
| --- | --- | --- | --- |
| **Erneute Dyspnoe**  **(nach Besserung)** | Nein  Ja, seit .. |  |  |
| **Tachypnoe nach Tag 3** | Nein  Ja, seit ..  Kein Effekt von Salbutamol | **SpO2 <90%**  **nach Tag 3** | Nein  Ja, seit ..  Kein Effekt von Salbutamol |
| **Fieber >38°C nach Tag 3** | Nein  Ja, bis .. | **Tod** | Nein  Ja, am .. |
| **Komplikationen der Pneumonie** | Nein  Ja, seit .. | ARDS  Pleuraerguss (Punktion)  Sepsis / Schock  Pleuraempyem  Lungenabszess  Andere_____________ | |
| **Andere Komplikationen** | Nein  Ja, seit .. | Otitis media  Meningo-Encephalitis  Andere:__________________________________ | |
| **Antibiotische Therapie wegen „Andere Komplikation“?** | Nein  Ja | von .. bis ..  mit __________________ | |
| **Intensivstation?** | Nein  Ja  von ..  bis .. | Beatmet für  Tage  Grund für IPS: | |
| **Gründe für Hospitalisation länger als IV AB Therapie (Total 100%)** | Medizinische ____%  (z.B. O_2_ Bedarf, Flüssigkeit)  Pflegerische __%  (z.B. professionelle Pflege nötig) | Elterliche %  (z.B. Wunsch/Sorge der Eltern)  Soziale %  (z.B. Austritt nach Hause/Heim nicht möglich) | |

| **Untersucher Initialen**  (Vor-/Nachnahme) | **Patienten-Nr.** | **Studien-Nr.** |
| --- | --- | --- |

**Procedere Tag 5**

**Prozedere**

**Impfstatus, falls nicht auf Tag 1 o. 3 CRF**

□ 1 x Pneumokokken □ 2 x Pneumokokken □ 3 x Pneumokokken □ 4 x Pneumokokken

□ 1 x HiB □ 2 x HiB □ 3 x HiB □ 4 x HiB

- **Blutentnahme (siehe Laborblatt Tag 5)**

**Gabe der Antibiotika gemäss ProCT (ng/ml):**

**□** >1 ug/L 7 days

**□** 0.51 - 1 ug/L 5 days

**□** 0.26 - 0.5 ug/L 3 days

**□** 0.1 - 0.25 ug/L stop AB

**□** <0.1 ug/L STOP AB

**Tagebuch prüfen und kopieren (AE?)**

**Overruling?**

- Kardio-Respir. Instabilität
- Schwere Sepsis/ Schock
- Therapiebeginn am Tag 3 oder 5

Rücksprache mit Basel bzw. Aarau

BS: 076 33 99 691 AR :076 33 99 694

□ **Guideline-Gruppe**

□ **Procalcitonin-Gruppe**

**Gabe der Antibiotika gemäss Guidelines:**

**□ keine antibiotische Therapie**

**□ Pneumonie (CAP):** - 5 – 10 Tage b. unkompl. Verlauf
 - > 14 Tage b. Erguss

- Gemäss Infektiol./Pneumol.: b. Abszess, Empyem

**□ Andere Gründe für AB: ______________________**

| **Bitte incl. Blatt „Tag 5“ sofort ans Datenzentrum faxen, vielen Dank: 061 685 60 12** |
| --- |

##

**Interview Tag 14**

## Appendix 6: CRF 4 –Day 14

| **Untersucher Initialen**  (Vor-/Nachnahme)) | **Patienten-Nr.** | **Studien-Nr.** |
| --- | --- | --- |

**Interview Tag 14, Datum** **.****.**(dd.mm.jj)

| **Bestehen heute noch Beschwerden bei Ihrem Kind?**  **­­** | Ja, welche? Seit wann?  ___________________________________  ___________________________________  ____________________________________  Nein |
| --- | --- |
| **War Ihr Kind hospitalisiert?** | Ja, für  Tage  Nein |
| **Ist Ihr Kind Ihrer Meinung nach zu früh entlassen worden?** | Ja, zu früh  Gerade richtig  Zu spät |
| **Wurde Ihrem Kind im Spital ein Antibiotikum verschrieben?** | Ja________________Einnahme?______________  Nein |
| **Wurde Ihr Kind nach dem** .. **(Tag 5) oder der Entlassung aus dem Krankenhaus** **nochmal von einem Arzt gesehen?** | Anzahl Termine:  mal im Studienspital  mal in anderem Spital:____________________  mal beim Kinderarzt:______________________ |
| **Warum wurde es gesehen?** | Gleicher Infekt:_____________________________  Erneuter Infekt:_____________________________  Anderer Grund_____________________________ |
| **Wurde erneut ein Antibiotikum verschrieben?** | Ja, welches_______________________________  Ja, genommen?____________________________  Nein |
| **Konnten Sie das Tagebuch für alle 14 Tage ausfüllen?** | Ja (🡪 Bitte schicken!)  Nein, weil_________________________________ |
| **Patient ist telefonisch nicht zu erreichen:**  **Versuche, Letzter Versuch am:** **.****.** | |
| **Bitte sofort ans Datenzentrum faxen, vielen Dank: 061 685 60 12** | |

## Appendix 7: Patient discharge information sheet

| **Untersucher Initialen**  (Vor-/Nachnahme) | **Patienten-Nr.** | **Studien-Nr.** |
| --- | --- | --- |

**Entlassungsinformation für Patienten**

**(Tag 5 bzw. bei Austritt)**

Sehr geehrte Dame / Sehr geehrter Herr

Nochmals vielen Dank für Ihre Bereitschaft, mit Ihrem Kind an unserer Studie zum gezielteren Einsatz von Antibiotika bei Infektionen der unteren Atemwege mit Hilfe des Blutmarkers Procalcitonin teilzunehmen.

**Was geschieht nun?**

Ihr Kind wurde in die  "Kontrollgruppe" bzw.  "Procalcitonin-Gruppe" eingeteilt. Entsprechend wurde bei ihr/ ihm die Indikation und Dauer der Antibiotikatherapie festgelegt:

Ihr Kind muss KEIN Antibiotikum (mehr) einnehmen.

Ihr Kind sollte das **Antibiotikum** bis am **.****.** weiter einnehmen.

Wir möchten im weiteren Verlauf sicherstellen, dass bei Kindern, bei denen die Antibiotikatherapie anhand des Blutswerts Procalcitonin gesteuert wurde, der Atemwegsinfekt gleich gut abheilt, wie bei Kindern ohne Procalcitonin-Messung. Deshalb bitten wir Sie, wie vereinbart, das **Tagebuch** weiter zu führen.

In 14 Tagen werden wir uns ein etwa 10-minütiges **Telefoninterview** mit Ihnen erlauben. Wir werden Sie dabei über bestehende **Beschwerden**, mögliche Rückfälle der Atemwegsinfektion und wie oft sie oft Sie nochmals einen Arzt oder das Spital aufsuchen mussten, befragen. Wir bitten Sie deshalb, auf diese Punkte zu achten und wenn nötig entsprechende **Notizen** zu machen.

Falls Ihr Kind sich nach Ende der Therapie plötzlich schlechter fühlt oder erneut Fieber oder vermehrt Atemnot und Husten bekommen sollte, sollten Sie unverzüglich unsere Notfallstation aufsuchen. Sie können in jedem Fall auch jederzeit mit uns Kontakt aufnehmen. Das Studienteam steht Ihnen selbstverständlich für Fragen und bei Unklarheiten zur Verfügung:

Für Ihre Bereitschaft, mit Ihrem Kind an der Studie teilzunehmen, bedanken wir uns herzlich - auch im Namen aller Kinder, die künftig bei ihrer Behandlung von den Ergebnissen dieser Studie profitieren können.

Das Studienteam

Kontaktadressen für Basel bzw. Aarau finden Sie auf Ihrem Tagebuch.

## Appendix 8: Office Paediatrician Information sheet

| **Untersucher Initialen**  (Vor-/Nachnahme) | **Patientenkleber** | **Studien-Nr.** |
| --- | --- | --- |

**Studieninformation für Ärzte**

Sehr geehrte Frau Kollegin / Sehr geehrter Herr Kollege

Ihre Patientin / Ihr Patient nimmt an der ProPAED-Studie teil, in der an mehreren Spitälern in der Schweiz der gezieltere Einsatz von Antibiotika bei Infektionen der unteren Atemwege studiert wird. Nachdem die Vorteile und die Sicherheit einer Procalcitonin (PCT) gesteuerten Strategie an über 1000 Erwachsenen am Universitätsspital Basel demonstriert werden konnten, soll dies nun bei Kindern gezeigt werden. Die Studie wurde von der lokalen Ethikkommission bewilligt und international registriert.

**Studienablauf und weiteres Prozedere**

Ihre Patientin / Ihr Patient wurde entweder in die nach momentanen Richtlinien behandelte

"Kontrollgruppe" oder in die  "Procalcitonin-Gruppe" randomisiert.

Entsprechend wurde bei ihr/ ihm die Indikation und Dauer der Antibiotikatherapie festgelegt:

Wir haben KEINE antibiotische Therapie duchgeführt.

PCT Kontrolle in den nächsten 24 Std und. ggf. Beginn einer AB Therapie.

Wir haben eine antibiotische Therapie durchgeführt. Das Ende der Therapie wird am Tag 3 oder 5 festgelegt.

Wir haben die Therapie am .. nach  Tagen gestoppt.

Wir bitten Sie die Antibiotikatherapie bis zum **.****.** weiterzuführen

Wir möchten im weiteren Verlauf sicherstellen, dass es bei beiden Patientengruppen zu einem gleich guten Abheilen des Atemweginfektes kommt und kein Therapieversagen aufgetreten ist. Deshalb werden wir im Rahmen der Studie eine **Nachkontrolle bei uns am Tag 3 und 5** durchführen und die Eltern Ihres Patienten nach 14 Tagen anrufen, um ein ca. 10-minütiges Telefoninterview durchzuführen.

Wenn es im Rahmen dieser Kontrollen Hinweise für ein Therapieversagen geben sollte, werden wir uns bei Ihnen melden. **Wir bitten Sie freundlich, das Studienteam im Falle eines Ihrerseits vermuteten Therapieversagens umgehend zu informieren.**

Falls Sie fragen zu dieser Studie haben, bitten wir Sie mit uns Kontakt aufzunehmen. Das Studienteam steht Ihnen gerne telefonisch zur Verfügung:

Mit bestem Dank und freundlichen, kollegialen Grüssen

Das Studienteam

Studienleitung:

Universitäts-Kinderspital beider Basel UKBB Prof. Dr. U. Heininger/ Dr. J. Bonhoeffer 076 33 99 691

**Bitte mit Kurzbericht sofort an den Kinderarzt faxen und Original in KG belassen!**

## Appendix 9: SAE report form to DSMB

| **Meldender Arzt**  **Vorname:**  **Nachname:** | **Patienten-Nr.** | **Studien-Nr.** |
| --- | --- | --- |

**Serious Adverse Event – Report Form**

**Geburtsdatum:** **.****.**(dd.mm.yy)

**Gruppenzuteilung:**  Kontroll - Gruppe  PCT – Gruppe

**Initiale Diagnose**:

**Datum Studieneinschluss**: **.****.**(dd.mm.yy)

**Ist initial eine Antibiotikagabe erfolgt?**  Ja Nein

**Antibiotikum**:       Dosis      mg/kg/d IVPO

**Wurde die initale AB Therapie gestoppt?**  Ja Nein

**.****.**(dd.mm.yy)

Grund:__________________________­­­­­­­­­­­_____________

**Datum des Adverse Events**:      

**Art des Adverse Events:**

Verlegung auf die Intensivstation Tod

Rezidiv des initialen Infektes Komplikationen des Infektes

Komplikationen der Antibiotikatherapie

**Kurze Problembeschreibung:**

**Bitte retournieren an Dr. med. Jan Bonhoeffer, Studienzentrum UKBB**

Fax: 061 685 60 12, jan.bonhoeffer@ukbb.ch

**Wichtige Telefon- und Faxnummern**

**Studienzentrum: Tel: 061 685 65 47 Dienstnatel Studienleitung Basel: 076 33 99 691**

**Fax: 061 685 60 12 Dienstnatel Studienleitung Aarau: 076 33 99 694**

## Appendix 10: DSMB decision form

| **Meldender Arzt**  **Vornahme:**  **Nachnahme:** | **Patienten-Nr.** | **Studien-Nr.** |
| --- | --- | --- |

**Serious Adverse Event - DSMB Statement:**

**Geburtsdatum:**

**Diagnose:**

**Beurteilung: Zusammenhang des Serious Adverse Events mit der ProPAED-Studie ?**

Sehr wahrscheinlich (**>75%**)

Wahrscheinlich (**50-75%)**

Unwahrscheinlich (**>25-50%**)

Sehr unwahrscheinlich (**<25%**)

**Modifikation des Studienprotokolls notwendig ?** Ja Nein

Falls “Ja”, welche ?

**Name des kommunizierenden DSMB Mitgliedes**:___________________________

Datum:_________________ Unterschrift: ________________

**Bitte retournieren an Dr. med. Jan Bonhoeffer, Studienzentrum UKBB**

**Fax: 061 685 60 12, jan.bonhoeffer@ukbb.ch**

## Appendix 11: Guideline for antibiotic treatment of pneumonia in children

**Guideline**

**zur antibiotischen Therapie der CAP bei Kindern**

Kinder mit milden Symptomen eines Infektes der unteren Luftwege brauchen keine antibiotische Therapie.

**Perorale Therapie**

• Die perorale antibiotische Therapie von Kindern mit CAP ist sicher und effektiv.

• Amoxicillin/Clavulansäure 80-90mg/kg/d in 2 ED ist das Mittel der Wahl bei Kindern unter 5 Jahren, da es gegen die meisten Erreger von CAP (inclusive intermediär resistenter Pneumokokken) in dieser Gruppe wirksam ist, gut vertragen wird und die Compliance optimiert. Clarythromycin 15mg/kg/d in 2 ED ist die empfohlene Alternative.

• Da Mycoplasma Pneumonien bei älteren Kindern deutlich häufiger sind, kann Clarythromycin in dieser Gruppe als erste Wahl gegeben werden.

• Bei V.a. auf Pneumonie durch Mycoplasmen oder Chlamydien ist Clarythromycin in jeder Altersstufe das Mittel der Wahl.

• Bei V.a. Pneumonie durch Pneumokokken ist Amoxicillin/Clavulansäure in jeder Altersstufe das Mittel der Wahl.

**Intravenöse Therapie**

• Die intravenöse Therapie sollte bei Kindern zur Anwendung kommen, bei denen eine perorale Therapie nicht möglich ist (z.B. rezidivierendes Erbrechen, zweifelhafte enterale Resorbtion) oder bei Kindern mit schwerer Symptomatik.

• Sinnvoll ist eine Therapie mit Amoxicillin/Clavulansäure 90mg/kg/d in 3 ED. Bei schweren Verläufen, kann eine weitere Dosis à 30mg/kg sinnvoll sein (120mg/kg/d). Bei septischen Kindern kann es sinnvoll sein ein 3. Generation Cephalosporin (z.B. Ceftriaxone 80-100mg/kg/d) einzusetzen.

• Sobald sich klinisch eine klare Besserung der Symptomatik einstellt, und die orale Einnahme gewährleistet ist, kann auf eine orale Therapie gewechselt werden.

## Appendix 12: Case scenarios of study flow

##

**PCT Group**

**PCT Group**

**T5**

**T14**

**T3**

**T1**

**AB**

**AB**

**AB**

**No AB**

**No AB**

**No AB**

**Standard**

**T1**

**T3**

**T5**

**T14**

**STOP AB**

**AB**

**PCT ↓**

**PCT ↑**

**STOP AB**

**AB**

**PCT ↓**

**PCT ↑**

**STOP AB**

**AB**

**PCT ↓**

**PCT ↑**

## Appendix 13: Laborauftragsformular

|  | **Patientenkleber** | **Studien-Nr.** |
| --- | --- | --- |

**Laborauftragsformular**

**Datum** **.****.**

**Venös (Standard): Tag 1: Tag 3: Tag 5:**

**400 μl Li-Heparin (PCT/CRP/ggf. Chemie) am Tag**

**4 ml EDTA (Studienplasma) am Tag**  **(+BB-diff)**

**Kapillär (Ausnahme!!!):**

**400 μl Li-Heparin (PCT/CRP/ggf. Chemie) am Tag**  **(2)**

**400 μl EDTA (Studienplasma) am Tag**

**Die gemessenen PCT Werte werden ausschliesslich via Studienwebsite (**[**http://propaed.pidb.ch**](http://propaed.pidb.ch/)**) mitgeteilt.**

**Interne Dokumentation des Labors:**

**Resultate:**

**PCT:___________μg/L**

**CRP:__________mg/L**

**IL-6: __________pg/ml**

**ADM:__________** **nmol/L**
